# Basal Primatomorpha colonized Ellesmere Island (Arctic Canada) during the hyperthermal conditions of the early Eocene climatic optimum

**DOI:** 10.1371/journal.pone.0280114

**Published:** 2023-01-25

**Authors:** Kristen Miller, Kristen Tietjen, K. Christopher Beard

**Affiliations:** 1 Biodiversity Institute, University of Kansas, Lawrence, Kansas, United States of America; 2 Department of Ecology and Evolutionary Biology, University of Kansas, Lawrence, Kansas, United States of America; Liverpool John Moores University, UNITED KINGDOM

## Abstract

Anthropogenically induced warming is transforming Arctic ecosystems across a geologically short timescale, but earlier episodes of Earth history provide insights on the nature and limitations of biotic change in a rapidly warming Arctic. Late early Eocene strata (~52 Ma) of the Margaret Formation on Ellesmere Island, Nunavut, Canada sample a warm temperate ecosystem with a polar light regime situated at ~77°N paleolatitude. This extinct boreal ecosystem hosted a diversity of early Cenozoic vertebrates, including thermophilic taxa such as crocodilians and tapiroid perissodactyls. Here we describe two new species of the early primatomorphan *Ignacius* from Ellesmere, which are by far the northernmost known records for Paleogene Primatomorpha. Ellesmere species of *Ignacius* are sister taxa, indicating a single colonization of Ellesmere from farther south in North America coincident with the onset of the hyperthermal Early Eocene Climatic Optimum (EECO). The Ellesmere *Ignacius* clade differs from closely related taxa inhabiting mid-latitudes in being larger (thereby conforming to Bergmann’s rule) and having modified dentition and muscles of mastication for a dietary regime emphasizing hard objects, possibly reflecting an increased reliance on fallback foods during long polar winters. The late early Eocene mammalian fauna of Ellesmere indicates that its unique paleoenvironment rendered it uninhabitable to some clades, including euprimates, while selected taxa were able to adapt to its challenging conditions and diversify.

## Introduction

**Fig 1:**
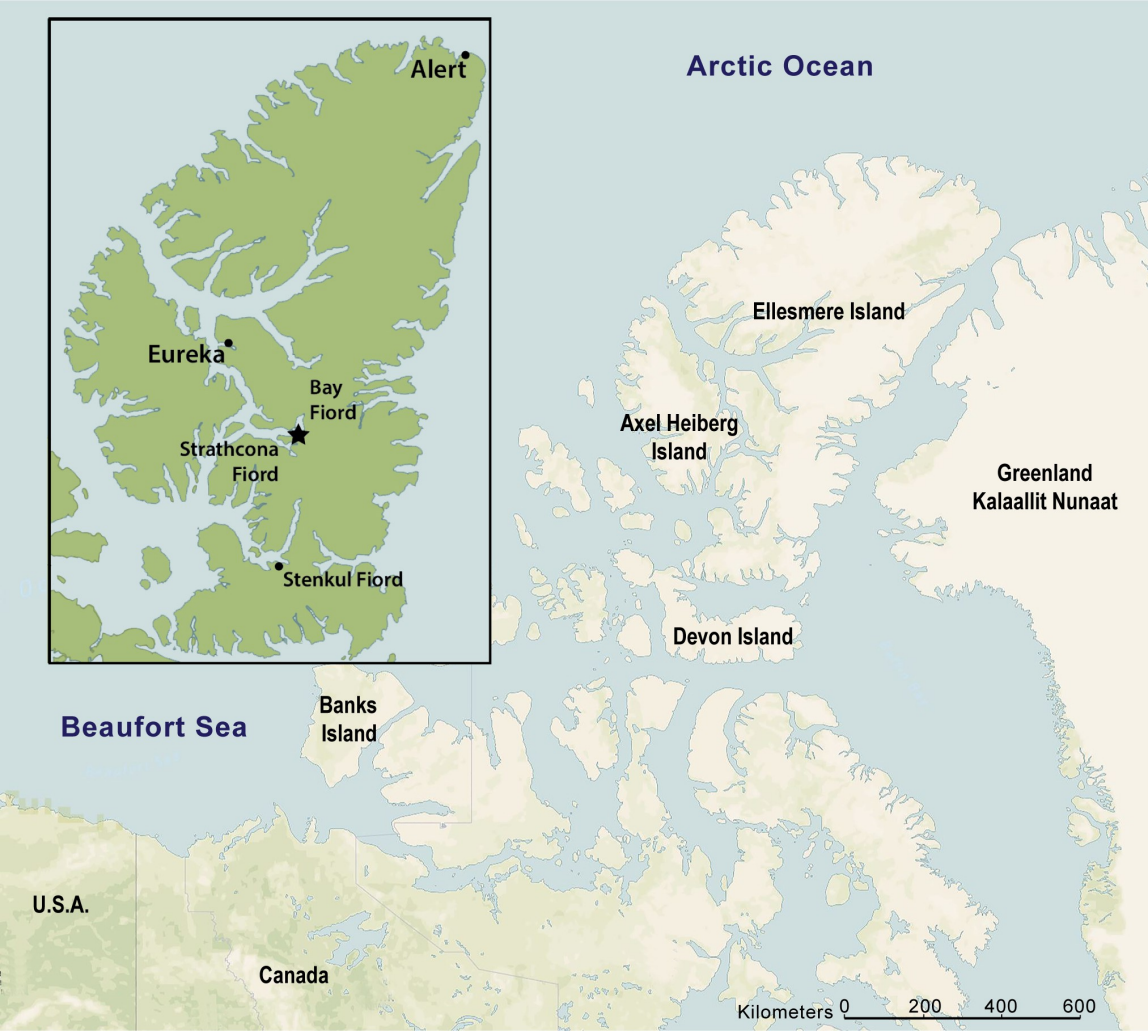
Map of Ellesmere Island, Nunavut, Canada and adjacent areas. The late early Eocene fossil sites yielding the paromomyid specimens described here are located on central Ellesmere Island near Bay Fiord (black star on enlarged map of Ellesmere shown as inset). Map adapted from public domain basemap available from U.S. Geological Survey (http://www.usgs.gov).

The discovery of fossil plants and vertebrates in Eocene rocks on Ellesmere and Axel Heiberg islands in the Canadian Arctic Archipelago has profoundly impacted efforts to model ancient climates and assess the paleobiology and paleobiogeography of early Cenozoic vertebrates living at high latitudes (Fig 1) [1–4]. During the greenhouse conditions of the early Eocene, Ellesmere hosted a warm temperate ecosystem comparable to modern cypress swamps of the southeastern United States, in tandem with a polar light regime consisting of roughly six months of winter darkness [3, 4]. Perhaps surprisingly, this extinct Arctic ecosystem was populated by a diversity of vertebrates, including such thermophilic taxa as the crocodilian *Allognathosuchus* [5]. The early Eocene (late Wasatchian) mammal fauna from Ellesmere broadly resembles contemporary faunas from lower latitudes of North America [6–14], although some significant differences in faunal composition exist. For example, as noted early in the history of research on the Eocene mammals of Ellesmere [2, 6], the diversity of plagiomenid mammals there is much greater than at lower latitudes of North America. In contrast, certain mammal clades that are well documented from lower latitudes are notably absent from the early Eocene of Ellesmere. Examples include artiodactyls, equid perissodactyls and *Hyopsodus*, the latter two of which are typically very abundant in early Eocene faunas from the North American Western Interior [15, 16].

Crown clade primates (or euprimates) are another group of Eocene mammals that have never been reported from Ellesmere, although they must have dispersed across high latitude regions such as Beringia to achieve their fully Holarctic distribution during the hyperthermal conditions of the Paleocene-Eocene Thermal Maximum [17]. Early Paleogene euprimates were diminutive in body size, invariably arboreal, and–like their modern relatives–among the most thermophilic of all mammals [17, 18]. If the absence of euprimates in the fossil record of Ellesmere accurately reflects their ancient distribution as opposed to being an artifact of sampling bias, the unique paleoenvironmental conditions prevailing at Ellesmere during the late early Eocene must have rendered it uninhabitable to this clade, despite its warm, wet and relatively equable climate. Indeed, the broad similarities in mammalian faunal composition between mid-latitude regions of North America and Ellesmere Island, encompassing taxa ranging from small microparamyine rodents to the cow-sized pantodont *Coryphodon* [7, 12], indicate an absence of obvious paleogeographic barriers to dispersal that might have prevented euprimates, artiodactyls, equids and *Hyopsodus* from colonizing Ellesmere.

Euprimates belong to a broader assemblage of thermophilic mammals known as Primatomorpha, which also includes dermopterans (also known as colugos or flying lemurs) as well as fossil taxa that are more closely related to primates and dermopterans than to other Euarchontoglires [19–22]. In addition to euprimates, several other clades of early Cenozoic Primatomorpha are recorded from the early Eocene of North America [23]. These taxa, often grouped as ‘plesiadapiforms,’ are variously considered as stem primates [23], stem dermopterans [24] or stem primatomorphans [25]. One of the plesiadapiform clades that persisted into the early Eocene, the family Paromomyidae, has previously been cited as occurring on Ellesmere [2, 3, 26], but the relevant fossils have never been formally studied or described. Here we describe two new species of paromomyids from Ellesmere based on fossil specimens collected over the course of several decades [1–3]. Because they are the only primatomorphans and the only apparently arboreal mammals currently documented from the Eocene of Ellesmere, the phylogenetic affinities, biogeographic history, and functional adaptations of Ellesmere paromomyids are important for constraining how and when early primatomorphans colonized the high Arctic and adapted to its challenging paleoenvironment. These data, in turn, illuminate the potential scope and inherent limitations of biotic change in a rapidly warming Arctic, insights that could be valuable as warmer climatic conditions transform Arctic ecosystems once again.

## Materials and methods

### Specimens

Eocene vertebrates from Ellesmere Island are derived from strata assigned to the Margaret Formation, Eureka Sound Group, which consists of coarsening upward cycles of interbedded sandstones, mudstones, and coals that are interpreted as representing proximal delta front to delta plain paleoenvironments [3, 14]. The paromomyid specimens that are the focus of this study were recovered from five localities within a late Wasatchian lower faunal level of the Margaret Formation near Bay Fiord on central Ellesmere Island (Fig 1) [14]. Paromomyid specimens from Ellesmere Island are permanently deposited in the Canadian Museum of Nature (**CMN**) in Ottawa, Ontario, Canada (S1 Table). Specimens were imaged using high-resolution microtomography (μCT) at the Museo Nacional de Ciencias Naturales in Madrid, Spain. μCT images and three-dimensional models of the Ellesmere paromomyid specimens are accessible through the online repository MorphoSource (S1 Table). Comparative specimens employed in this analysis are permanently deposited in the following repositories: **AMNH,** American Museum of Natural History, New York, New York, USA; **CM,** Carnegie Museum of Natural History, Pittsburgh, Pennsylvania, USA; **KUVP**, Division of Vertebrate Paleontology, Biodiversity Institute, University of Kansas, Lawrence, Kansas, USA; **PAT**, Université de Montpellier (Palette collection), Montpellier, France; **UALVP**, University of Alberta Laboratory of Vertebrate Paleontology, Edmonton, Alberta, Canada; **UCM**, University of Colorado Museum of Natural History, Boulder, Colorado, USA; **UM**, Museum of Paleontology, University of Michigan, Ann Arbor, Michigan, USA; **USNM**, National Museum of Natural History, Smithsonian Institution, Washington, DC, USA; **UWBM,** Burke Museum, University of Washington, Seattle, Washington, USA; **YPM-PU,** Princeton University Collection housed at Yale Peabody Museum, Yale University, New Haven, Connecticut, USA.

### Nomenclatural acts

The electronic edition of this article conforms to the requirements of the amended International Code of Zoological Nomenclature (ICZN), and hence the new names contained herein are available under that Code from the electronic edition of this article. This published work and the nomenclatural acts it contains have been registered in ZooBank, the online registration system for the ICZN. The ZooBank LSIDs (Life Science Identifiers) can be resolved and the associated information viewed through any standard web browser by appending the LSID to the prefix http://zoobank.org/. The LSID for this publication is: urn:lsid:zoobank.org:pub:630C54BC-33D4-46CC-ADBC-7607D36B785E. The electronic edition of this work was published in a journal with an ISSN, and has been archived and is available from the following digital repositories: PubMed Central, LOCKSS.

### Dental measurements and terminology

Standard dental measurements were obtained using digital Mitutoyo micrometers paired with a measuring stage under a Unitron Z6 binocular microscope equipped with an ocular reticle. Dental terminology follows the nomenclature of Ni et al. [25].

### Phylogenetics

We augmented a character-taxon matrix originally developed to assess the phylogenetic relationships of European paromomyids [27] by adding seven additional taxa (including all known species of *Ignacius*) and ten additional characters. *Torrejonia sirokyi* was selected as an additional outgroup because it represents an early member of the Palaechthonidae, a family often thought to be closely related to paromomyids [23, 28]. *Chronolestes simul* was chosen as an additional outgroup because it is thought to be a relatively primitive plesiadapoid [29, 30]. With one exception, we retained the coding scheme established for the prior version of this matrix [27]. Based on the morphology of M_2_ in UWBM 97705, we rescored character 44 for *Paromomys farrandi* from state “0” (v-shaped protocristid) to state “1” (slightly concave protocristid). The list of characters (S2 Table), character-taxon matrix (S1 File), and coding scheme are included in supplementary information. All characters were treated as unweighted and most characters were treated as unordered. Several multistate characters that conform to natural morphoclines (characters 1, 10, 16, 22, 28 and 50) were treated as “ordered” or “additive” for purposes of parsimony analysis. Parsimony analyses were performed with the phylogenetic software TNT [31] through a traditional heuristic search using 1000 random addition sequences as starting trees and tree bisection reconnection branch swapping with 100 trees saved per replication.

In an effort to incorporate stratigraphic data within our phylogeny, we compiled first appearance data for each of the taxa in our matrix (S3 Table). We then used the R package, strap (Stratigraphic Tree Analysis for Palaeontology) [32] to produce a time-scaled phylogeny using the DatePhylo function with a root length of one and method set to “equal.” Because most of the taxa included in our phylogeny are known from a single time interval, we used the same dates for first and last appearances when importing the age data into R. The “equal” method of scaling phylogenies avoids branches of zero length by equally distributing branch lengths along the tree. Accordingly, this method does not necessarily estimate divergence dates accurately. The geoscalePhylo function was used to plot the time-scaled phylogeny against the geological time scale.

### Dental topography

With the advent of modern μCT technologies that enable the routine acquisition of highly resolved surface meshes of teeth, algorithms for quantifying three-dimensional tooth shape have been developed and applied to living and fossil primatomorphans and other mammals [33–39]. Three commonly used metrics for quantifying dental topography are: relief index (RFI), which captures the ratio between the 2D area and 3D area of a tooth, thereby quantifying the relative height of the tooth crown; orientation patch count rotated (OPCR), which captures the complexity of a tooth surface; and Dirichlet normal energy (DNE), which quantifies the surface curvature [35]. In our study, we chose to use ariaDNE (a robustly implemented algorithm for Dirichlet normal energy) in place of DNE [36]. ariaDNE quantifies surface curvature but is less sensitive to mesh preparation procedures than DNE [36]. Our dental topographic comparisons are based on μCT scans of lower molars of eight species of paromomyids and an extant data set of 95 lower second molars representing 11 genera of platyrrhines [33]. In our data set, we replaced the platyrrhine DNE values from Winchester et al. [33] with ariaDNE values of the same specimens from Shan et al. [36]. μCT data were rendered, molars individually sectioned, cropped at the root-crown junction, and saved as.ply files using Mimics (v. 24.0). The resulting mesh for each tooth was then pre-processed following Prufrock et al. [34]. ariaDNE was calculated for the fossil specimens using the ariaDNE script in MATLAB (R2021a) [36] with bandwidth set to 0.08. OPCR and RFI were calculated using the R package molaR with RFI alpha set to 0.067 and no changes made to the default arguments for the OPCR function [37].

### Statistical analyses

All statistical analyses were performed in R v4.1.1. To assess the probable dietary adaptations of fossil taxa, a principal component analysis (PCA) was performed using a combined dataset of extant platyrrhines [33] and fossil paromomyids with RFI, ariaDNE, and natural log of molar length as variables. We then used the results from the PCA to perform a MANOVA, testing for differences between the extant dietary groups and the fossil taxa. Based on our phylogenetic analysis (see below) and the results from the PCA, the fossil taxa were partitioned into Arctic and mid-latitude groups for the MANOVA. Significant MANOVA results were then followed up with ANOVAS for each principal component and post hoc pairwise comparisons using Tukey’s Honest Significant Difference (HSD) tests. We omitted OPCR as a variable in our PCA because the methodology used to calculate OPCR in the platyrrhine comparative dataset [33] produces values that are not strictly comparable to OPCR values obtained using molaR [37]. However, we did use OPCR to explore variation in surface complexity among the fossil taxa. Data and code used for the PCA and MANOVA are available in supplementary information as S3–S5 Files.

To test for differences between dental topography metrics among paromomyid species, a one-way ANOVA was performed for each topographic metric (RFI, OPCR, and ariaDNE). Post hoc pairwise comparisons were then performed using Tukey’s Honest Significant Difference test. In an attempt to understand the dietary differences between paromomyids living at different latitudes, we maintained the partition between Arctic and mid-latitude paromomyids. We then performed two-sided, equal variance t-tests to test for significant differences in lower molar dental topography metrics between the geographically distinct samples. Data and code used for the ANOVAs are available in supplementary information as S2 and S5 Files.

## Results

### Systematic paleontology

Mammalia Linnaeus, 1758 [40]

Placentalia Owen, 1837 [41]

Primatomorpha Beard, 1991 [42]

Paromomyidae Simpson, 1940 [43]

*Ignacius* Matthew and Granger, 1921 [44]

#### Type species

*Ignacius frugivorus* Matthew and Granger, 1921 [44].

#### Included species

*Ignacius frugivorus* Matthew and Granger, 1921 [44]; *Ignacius fremontensis* (Gazin, 1971) [45]; *Ignacius graybullianus* Bown and Rose, 1976 [46]; *Ignacius clarkforkensis* Bloch et al. 2007 [28].

#### Remarks

*Ignacius* is a genus of Paromomyidae that for many years was considered to be a synonym of the closely related *Phenacolemur* [46]. *Ignacius* has previously been documented from Paleocene and early Eocene strata across much of western North America (specifically California, Texas, Colorado, Wyoming, Montana, North Dakota, Alberta and Saskatchewan) [28, 44–55]. The oldest known record of *Ignacius* occurs at Rock Bench Quarry (late Torrejonian) in the northern Bighorn Basin, Wyoming [53]. The youngest well-documented records of *Ignacius* are reported here from the late early Eocene of Ellesmere Island (Arctic Canada).

*Ignacius mckennai*, sp. nov. urn:lsid:zoobank.org:act:C399F91D-AD55-48F7-BB10-6B797645DE15

#### Holotype

**Fig 2:**
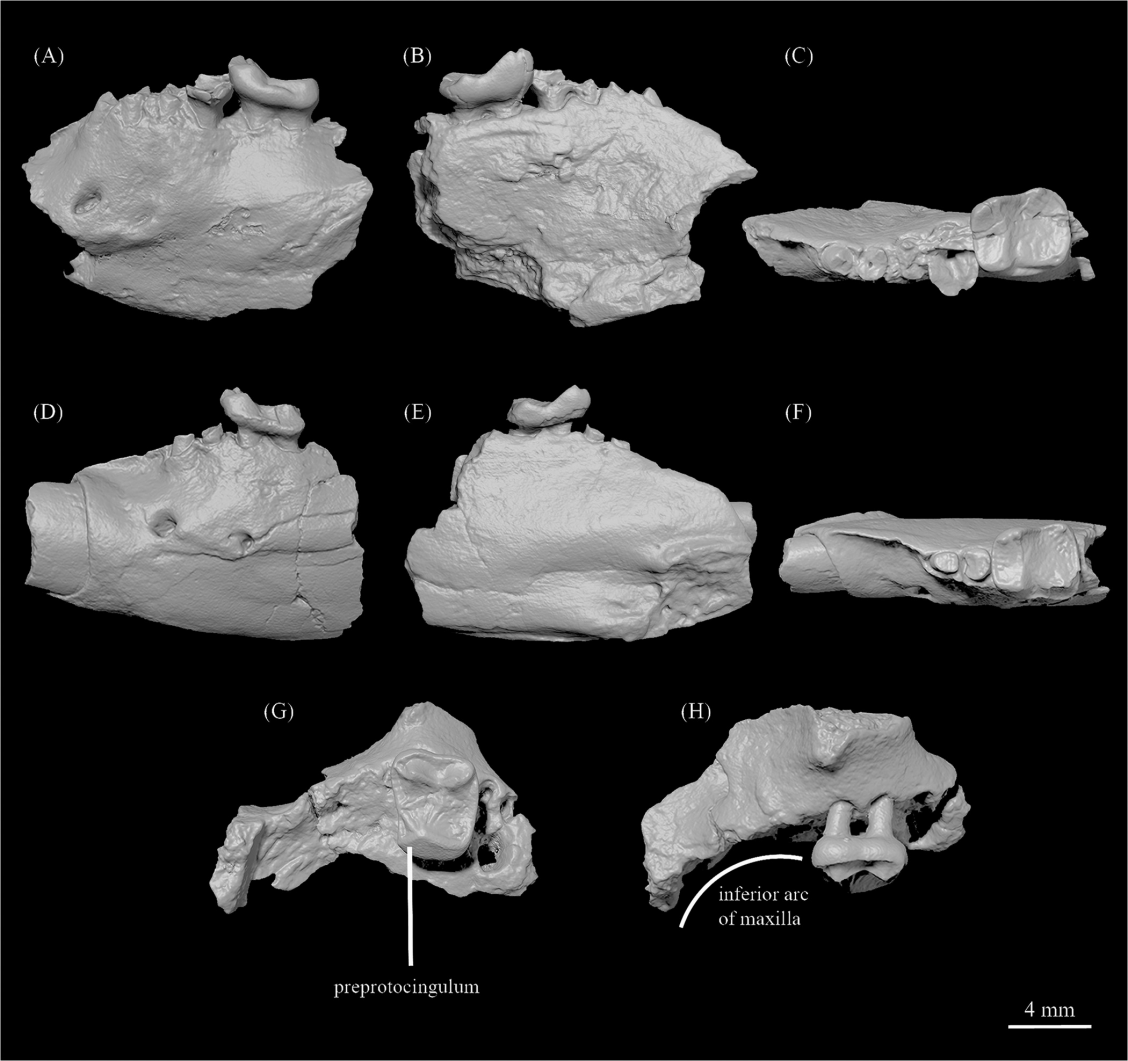
Dentition of *Ignacius mckennai* sp. nov. from Ellesmere Island, Nunavut, Canada. **A-C,** CMN 30850, left dentary fragment with M_2_ in buccal (**A**), lingual (**B**), and occlusal (**C**) views; **D-F,** CMN 30986, left dentary fragment with M_1_ in buccal (**D**), lingual (**E**), and occlusal (**F**) views; **G, H**, CMN 30830 (holotype), left maxillary fragment with M^1^ in occlusal (**G)** and buccal (**H**) views.

CMN 30830, left maxillary fragment preserving M^1^ and the alveoli for P^3-4^ and M^2^ (Fig 2G and 2H).

#### Paratypes

CMN 30850, left dentary fragment with the alveolus of I_1_, roots of P_4_-M_1_, and crown of M_2_ (Fig 2A–2C); CMN 30986, left dentary fragment with roots of I_1_ and P_4_ and crown of M_1_ (Fig 2D–2F).

#### Diagnosis

Largest known species of *Ignacius*. Differs from *I*. *fremontensis* in lacking P_3_. Further differs from *I*. *fremontensis*, *I*. *frugivorus*, *I*. *clarkforkensis* and *I*. *graybullianus* in having crenulated molar enamel, M^1^ with broader buccal cingulum and neomorphic crest or preprotocingulum linking protocone with mesiolingual cingulum, and M_1-2_ with stronger buccal cingulids. Origin of zygomatic process more rostral than in other species of *Ignacius* except *I*. *graybullianus*.

#### Type locality

ELS locality 76–85, central Ellesmere Island, Nunavut, Canada.

#### Age and known distribution

The type locality and ELS locality 76–56, both of which are included in the lower faunal level of the Margaret Formation, Eureka Sound Group, Ellesmere Island. Biostratigraphic and geochronological data indicate that these localities are latest Wasatchian in age, falling within the early part of the EECO [3, 14].

#### Etymology

Named in honor of Malcolm C. McKenna, in recognition of his contributions to the geology and mammalian paleontology of Ellesmere Island [6, 8, 9, 26].

#### Description and comparisons

Metric data for the current sample are provided in Table 1. As noted in the diagnosis, *I*. *mckennai* is the largest known species of *Ignacius*, being 55–82% larger in linear dental measurements than *I*. *clarkforkensis*, which is the largest species of *Ignacius* described from mid-latitudes of North America. *I*. *mckennai* is also larger than the other new species of *Ignacius* from Ellesmere Island (described below), with linear dental measurements that range from 15–42% larger in *I*. *mckennai*.

**Table 1:** Summary of dental measurements (in mm) for *Ignacius mckennai* from Ellesmere Island, Nunavut, Canada.

Specimen #	Tooth Locus	Width	Length
CMN 30830	M^1^	5.14	4.11
CMN 30986	M_1_	3.53	4.26
CMN 30850	M_2_	4.00	4.68

**Fig 3:**
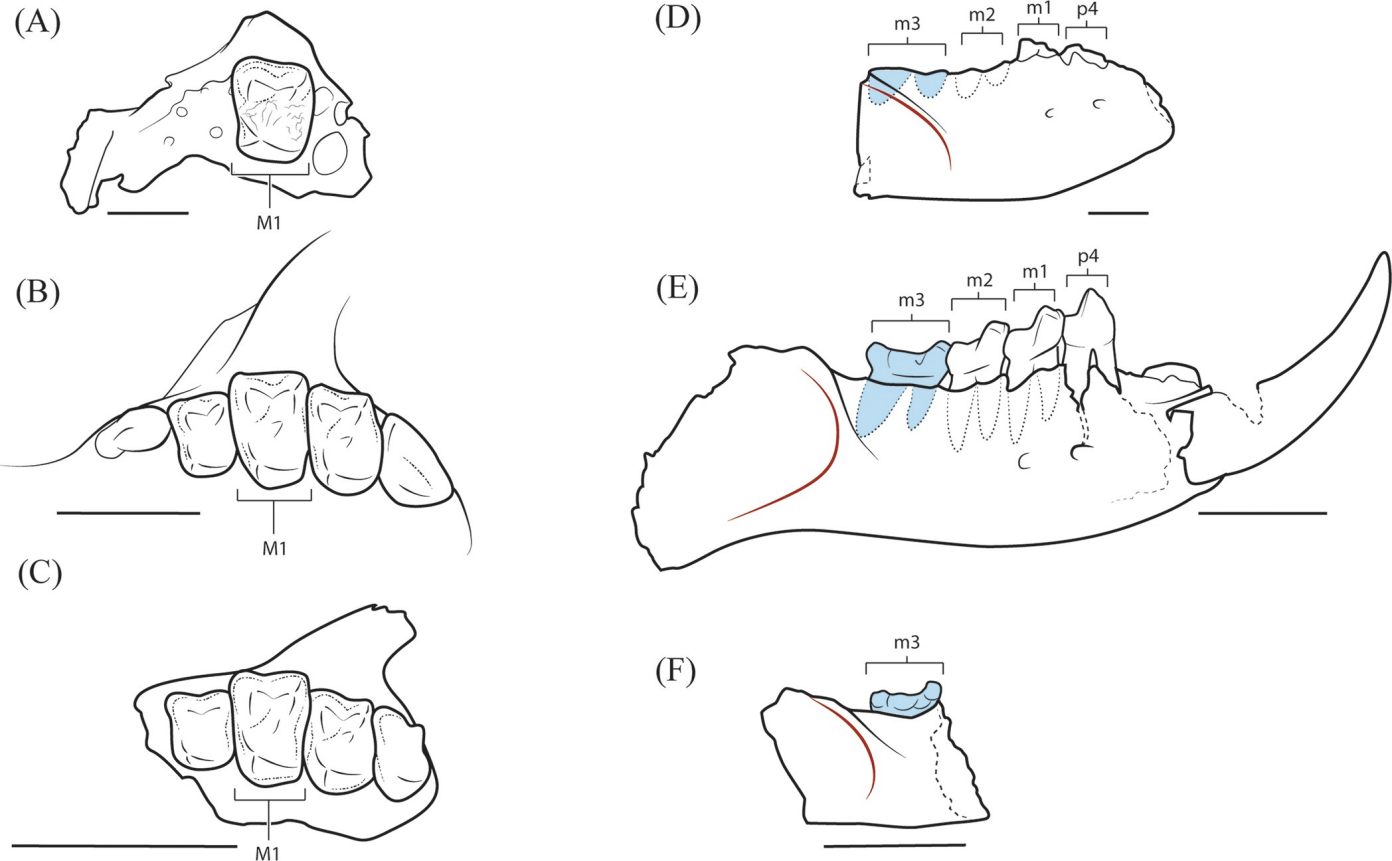
Bony landmarks delimiting the muscles of mastication in selected paromomyids. **A-C,** Position and orientation of the zygomatic root in *Ignacius mckennai* (CMN 30830) (**A**), *Ignacius graybullianus* (USNM 421608) (**B**), and *Ignacius fremontensis* (CM 77005) (**C**), showing the rostral displacement of the zygomatic (above M^1^) in *I*. *mckennai* and *I*. *graybullianus* relative to the condition in *I*. *fremontensis* (above M^2^). **D-E**, Position of the anterior margin of the masseteric fossa (indicated by red line) in *Ignacius dawsonae* (CMN 30853) (**D**), *Ignacius clarkforkensis* (UM 108210) (**E**), and *Ignacius fremontensis* (CM 86602) (**F**), showing the anterior extension of the masseteric fossa (below the level of interstitial contact between M_2_ and M_3_) in *I*. *dawsonae*. Scale bars = 4mm.

Aspects of maxillary morphology are revealed by the holotype. In CMN 30830 (Fig 2G and 2H), the root of the zygomatic process originates above M^1^, which is more rostral than in other species of *Ignacius* aside from *I*. *graybullianus* (Fig 3A–3C). The anteroventral margin of the zygomatic process is swollen to form a small tuberosity for the origin of the superficial masseter. The infraorbital foramen is relatively large (1.54 mm mediolaterally; 2.26 mm dorsoventrally) and positioned directly superior to the mesial alveolus of P^3^. The anterior part of the maxilla, occupying the diastema between the anterior dentition and P^3^, arcs inferiorly. This part of the maxilla is more nearly straight in *I*. *graybullianus* [54, 55].

M^1^ is brachyodont and nearly square in occlusal outline (Fig 2G and 2H), in contrast to the more transverse upper molar proportions found in other species of *Ignacius* aside from *I*. *graybullianus*. The paracone and metacone are almost equal in height, with the metacone being only slightly shorter than the paracone. A strong buccal cingulum is present, which becomes continuous with the postmetacrista at the distobuccal margin of the tooth. A short, weakly defined preparacrista is present. The postparacrista and premetacrista are not as obliquely oriented as in *I*. *clarkforkensis* and *I*. *graybullianus*, but more oblique than in *I*. *frugivorus* and *I*. *fremontensis*. The postprotocrista is present but relatively weak, so that the trigon and posterolingual basins are nearly continuous. A short neomorphic crest, designated here as the preprotocingulum (Fig 2G), connects the protocone to the mesiolingual cingulum. The preprotocrista runs mesiobuccally from the protocone, becoming continuous with the mesial cingulum near the site where a weak crest that may be homologous with the postparaconule crista diverges toward the apex of the paracone. A second weak crest runs more distally from the buccal terminus of the preprotocrista toward the junction between the postparacrista and the premetacrista. Distinctly cuspate paraconule and metaconule are absent, although minor swelling on the postprotocrista may mark the location of a vestigial metaconule. Overall crown topography is low and flat, so that the trigon and the adjacent posterolingual basin are nearly continuous, being demarcated by a relatively weak postprotocrista. In other species of *Ignacius*, the trigon is elevated with respect to the posterolingual basin. Because of the nearly square occlusal outline of M^1^, the postprotocingulum is relatively longer than it is in *I*. *fremontensis* and *I*. *frugivorus*.

Both CMN 30850 (Fig 2A–2C*)* and CMN 30986 (Fig 2D–2F) preserve aspects of the dentary and lower dentition. Two mental foramina are present beneath the mesial roots of P_4_ and M_1_, respectively. The lower dental formula is 1-0-1-3, although several lower tooth loci are only represented by roots and/or alveoli. Based on the root of I_1_ as preserved in CMN 30986, the lower central incisor was large, procumbent, and nearly horizontal in orientation (Fig 2D). The crown of P_4_ remains unknown, but this tooth was double-rooted. A strong dorsal crest spans the diastema between I_1_ and P_4_ (Fig 2F). The symphysis (best preserved in CMN 30986) is unfused and procumbent but robustly constructed, with a pit for origin of genioglossus separating medially projecting bony shelves that are homologous to the superior and inferior transverse tori of taxa having fused symphyses (Fig 2E).

While neither dentary preserves the crown of P_4_, the short length (~2.6 mm) spanning the P_4_ roots suggests a relatively compressed P_4_ crown, in contrast to the longer and narrower P_4_ crowns of *I*. *fremontensis* and *I*. *frugivorus*. M_1-2_ are brachyodont and relatively homodont, with trigonids that are inclined mesially, making them only modestly taller than their talonids. While M_1-2_ are generally similar, the trigonid of M_1_ is mesiodistally longer and buccolingually narrower than that of M_2_. On both M_1-2_ the paraconid and metaconid are closely appressed, but the paraconid is taller and more voluminous than the metaconid, especially on M_2_ (CMN 30850). Additionally, the protoconid and metaconid are widely spaced, resulting in a broad and relatively flattened trigonid. The cristids obliquae on M_1-2_ are oriented relatively mesiodistally, yielding weakly invaginated hypoflexids. Buccal cingulids are well developed, particularly near the junction between talonid and trigonid. Molar enamel is moderately crenulated.

*Ignacius dawsonae*, sp. nov. urn:lsid:zoobank.org:act:842621DC-E6DB-418F-B575-C638473334B2

#### Holotype

**Fig 4:**
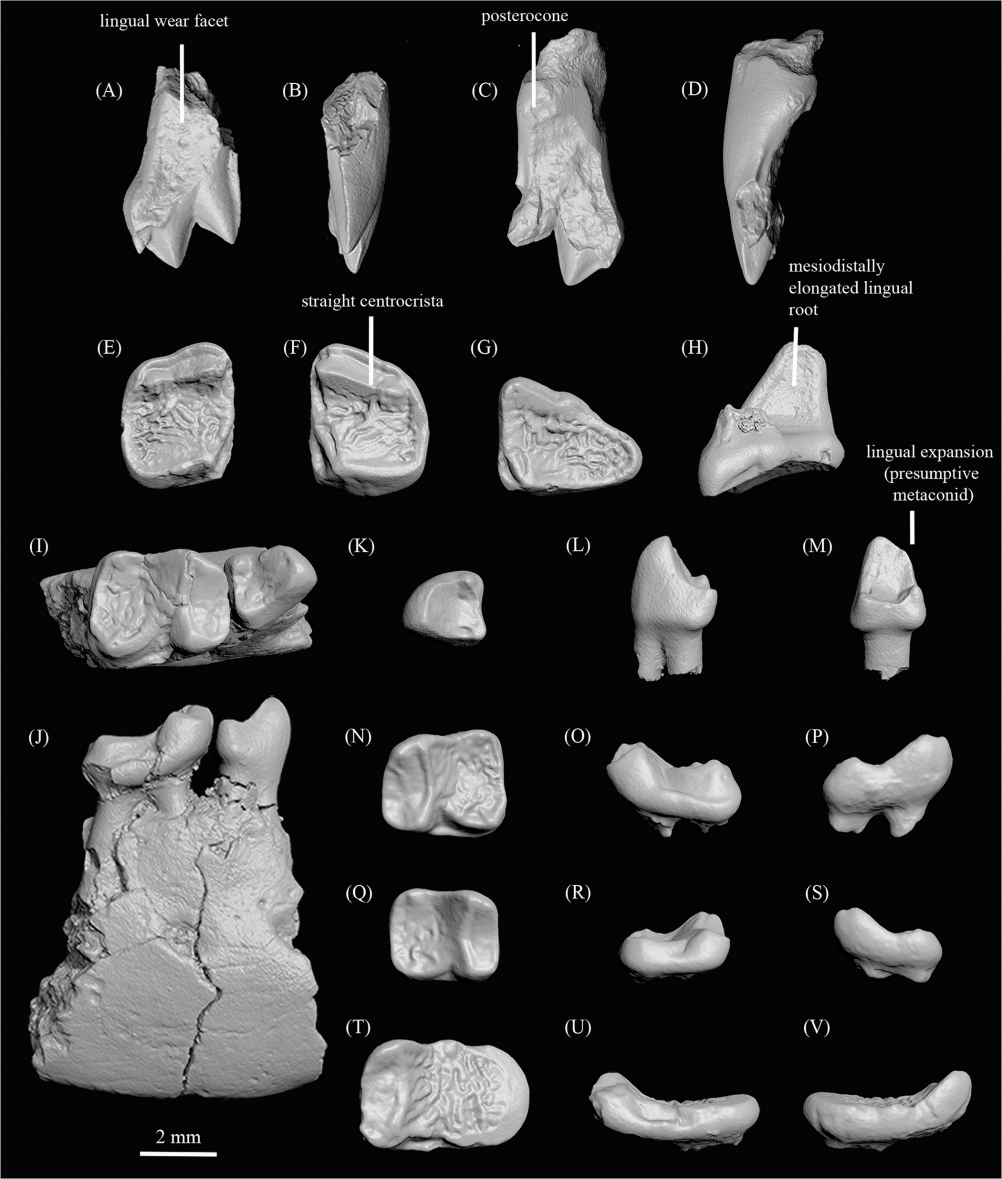
Dentition of *Ignacius dawsonae* sp. nov. from Ellesmere Island, Nunavut, Canada. **A, B,** CMN 32325, apical fragment of right I^1^ in lingual (**A**) and distal (**B**) views; **C, D,** CMN 30903, left I^1^ in lingual (**C**) and distal (**D**) views; **E,** CMN 32320, right M^1^ in occlusal view; **F,** CMN 30868, left M^2^ in occlusal view; **G, H,** CMN 32321, left M^3^ in occlusal (**G**) and buccal (**H**) views; **I, J,** CMN 30837 (holotype), right dentary fragment with P_4_-M_1_ in occlusal (**I**) and buccal (**J**) views; **K-M,** CMN 30949, left P_4_ in occlusal (**K**), buccal (**L**), and distal (**M**) views; **N-P,** CMN 30999, left M_1_ in occlusal (**N**), buccal (**O**), and lingual (**P**) views; **Q-S,** CMN 30856, right M_2_ in occlusal (**Q**), buccal (**R**), and lingual (**S**) views; **T-V,** CMN 30889, left M_3_ in occlusal (**T**), buccal (**U**), and lingual (**V**) views.

CMN 30837, right dentary fragment preserving P_4_-M_1_ (Fig 4I and 4J).

#### Paratypes

CMN 30828, right M_1_; CMN 30831, left dentary fragment preserving roots for M_2_ and talonid fragment of M_3_; CMN 30835, left edentulous dentary fragment preserving alveoli for P_4_-M_2_; CMN 30853, right edentulous dentary fragment preserving roots for I_1_-M_1_ and alveoli for M_2-3_; CMN 30856, right M_2_ (Fig 4Q–4S); CMN 30864, left dentary preserving M_2_; CMN 30867, fragmentary left M_2_; CMN 30868, left M^2^ (Fig 4F); CMN 30883, fragmentary left M_3_; CMN 30889, left M_3_ (Fig 4T–4V); CMN 30902, fragmentary right M_3_; CMN 30903, left I^1^ (Fig 4C and 4D); CMN 30927, right M_3_ talonid fragment; CMN 30933, fragmentary left M_3_; CMN 30936, left P_4_; CMN 30949, left P_4_ (Fig 4K–4M); CMN 30954, left M_1_; CMN 30959, right M_1_; CMN 30988, left dentary fragment preserving roots of M_2_ and talonid of M_3_; CMN 30995, right M^1^; CMN 30996, right M^2^; CMN 30997, left M_3_; CMN 30998, right M^2^; CMN 30999, left M_1_ (Fig 4N–4P); CMN 32320, right M^1^ (Fig 4E); CMN 32321, left M^3^ (Fig 4G and 4H); CMN 32325, apical part of right I^1^ (Fig 4A and 4B).

#### Diagnosis

Differs from other species of *Ignacius* in having upper molars with lower, less cuspate paracone and metacone more nearly integrated within a relatively straight, trenchant centrocrista. Differs from other species of *Ignacius* except *I*. *mckennai* in having upper molars with well-defined preprotocingulum and crenulated enamel. Roughly 40% smaller than *I*. *mckennai* and with stronger enamel crenulation. M^3^ talon more expanded than in other *Ignacius* species. Trigonid of P_4_ broader than in other species of *Ignacius*, with neomorphic lingual expansion of protoconid and associated postvallid crest. Lower molars with stronger buccal cingulids, broader trigonids, and paraconid and metaconid more closely connate than in *I*. *fremontensis* and *I*. *frugivorus*. M_3_ with low trigonid and relatively flat talonid lacking distinct cusps, in contrast to other species of *Ignacius*. Masseteric fossa of dentary extends farther anteriorly than in other species of *Ignacius* (with the possible exception of *I*. *mckennai*, in which this feature remains undocumented) (Fig 3D–3F).

#### Type locality

ELS locality 76–85, central Ellesmere Island, Nunavut, Canada.

#### Age and known distribution

The type locality and ELS localities 76–44, 76–49, 76–56, and 76–84, all of which are included in the lower faunal level (late Wasatchian) of the Margaret Formation, Eureka Sound Group, Ellesmere Island [3, 14]. S1 Table provides data on the provenance of all paromomyid specimens currently known from Ellesmere Island.

#### Etymology

Named in honor of Mary R. Dawson, in recognition of her pioneering research on the geology and mammalian paleontology of Ellesmere Island [1, 2, 6, 7, 12, 13].

#### Description and comparisons

Metric data for the current sample are provided in Table 2. Two upper central incisors of *I*. *dawsonae* are known, both of which show an unusual wear pattern, in which the apices of the anterocone and laterocone are relatively pristine, while the lingual surface of the crown is heavily worn (Fig 4A–4D). I^1^ is dominated by the anterocone and laterocone, which are labiolingually compressed with sharp crests extending mesially and distally from their apices. A deep, V-shaped cleft divides the bases of the anterocone and laterocone. The apex of the laterocone projects distally away from the anterocone and is positioned more apically than in *I*. *fremontensis* and *I*. *frugivorus*. A small but relatively pyramidal mediocone is present near the mesial base of the anterocone. A mediocrista is present, but its degree of development and full lingual extent are impossible to determine because of heavy lingual wear. A flat interstitial wear facet for the contralateral I^1^ is present near the mesial base of the mediocone. Wear obscures the morphology of the posterocone, but it appears not to have been very voluminous (Fig 4C).

**Table 2:** Summary of dental measurements (in mm) for *Ignacius dawsonae* from Ellesmere Island, Nunavut, Canada.

Tooth Locus	n	Length				Width			
		x	OR	s	V	x	OR	s	V
I^1^	2	2.29	2.07–2.52	0.32	13.95	3.50	3.29–3.71	0.30	8.58
M^1^	3	3.19	3.11–3.30	0.10	3.10	3.91	3.74–4.03	0.15	3.84
M^2^	2	3.80	3.32–4.28	0.68	17.89	3.86	3.54–4.18	0.45	11.66
M^3^	1	-	4.21	-	-	-	3.08	-	-
P_4_	3	2.26	2.20–2.36	0.08	3.54	2.09	2.00–2.29	0.17	8.13
M_1_	5	3.43	3.04–3.65	0.26	7.58	3.03	2.85–3.24	0.15	4.95
M_2_	2	3.30	2.93–3.67	0.52	15.76	2.90	2.56–3.23	0.47	16.21
M_3_	2	5.07	5.00–5.14	0.09	1.78	3.19	3.09–3.37	0.11	3.45

**Abbreviations: n,** number of specimens; **x**, mean; **OR**, observed range; **s**, standard deviation; **V**, coefficient of variation.

M^1-2^ of *I*. *dawsonae* are buccolingually compressed and relatively square in occlusal outline (Fig 4E and 4F). The paracone and metacone are low and weakly cuspate, being integrated within a linear and relatively trenchant centrocrista that contrasts with the obliquely oriented postparacrista and premetacrista found in other species of *Ignacius* [28, 46]. There is no distinct postprotocrista, resulting in continuity between the trigon and the posterolingual basin, both of which are adorned by strong enamel crenulation. The postprotocingulum is long, elevated, and continuous with the raised distal cingulum defining the distal margin of each upper molar. M^3^ is notable for the extreme reduction of its trigon cusps, none of which are distinctly cuspate (Fig 4G and 4H). The paracone and metacone are greatly reduced and fully incorporated within a mesiodistally straight centrocrista, which forms a crest along the mesiobuccal side of the crown. The protocone is slightly more distinct than the paracone and metacone, but it is also integrated within a raised and mesiodistally extensive postprotocingulum. The latter structure is continuous with a raised distal cingulum, which arcs around the distobuccal margin of the tooth before essentially fusing with the centrocrista mesially. Because of the great length of the postprotocingulum, a mesiodistally expanded talon basin occupies roughly half the areal extent of the entire crown. The postprotocrista is absent, so that the trigon and talon are confluent, shallowly basined, and adorned with extremely crenulated enamel. A short preprotocrista merges with a raised mesial cingulum, which joins the centrocrista near the mesiobuccal corner of the tooth. In general, the M^3^ crown forms a shallowly concave and highly crenulated surface that is surrounded on all sides by raised crests or cingula. A short precingulum is restricted to the mesiolingual side of the crown, extending from the level of the protocone to the junction between the preprotocrista and the raised mesial cingulum. The lingual root of M^3^ is greatly expanded mesiodistally, running almost the entire length of the crown, matching the expansion of the postprotocingulum and talon basin. In contrast, the two buccal roots are small, closely spaced or even partly fused, and restricted to the mesial part of the tooth.

The dentary, best preserved in CMN 30853, is notable in having an anteriorly expanded masseteric fossa, so that the crest or ridge marking the superior margin of the masseteric fossa extends below the level of interstitial contact between M_2_ and M_3_. In other species of *Ignacius*, the masseteric fossa is less expansive and typically extends no farther than the talonid of M_3_ (Fig 3D–3F).

P_4_ is remarkably short and broad, and its two roots are very closely spaced if not partly fused (Fig 4L). The protoconid is buccolingually broad, and its distal surface forms the vertically oriented postvallid. Weak crests occur on either side of the postvallid, which meet at the apex of the protoconid. The buccal postvallid crest is confluent with the mesiodistally short cristid obliqua. As a result, the buccal side of the crown continues uninterrupted from the trigonid to the talonid, leaving no space for a hypoflexid. The lingual postvallid crest and the lingual side of the protoconid bulge lingually, where a tiny swelling of enamel could be considered a presumptive metaconid. The mesial side of the protoconid is smoothly rounded, with no development of a mesial protoconid crest or paracristid. The talonid is mesiodistally short but wide, like that of *I*. *graybullianus*. The entoconid is relatively tall and cuspidate, while the hypoconid is shorter and blunt. Minor enamel crenulation occurs on the postvallid and in the talonid basin.

The trigonids of M_1_ and M_2_ are mesiodistally short and broad, with paraconid and metaconid closely connate and a low protoconid. On relatively unworn specimens such as CMN 30828 and CMN 30999 (Fig 4N–4P), a low but distinct protocristid runs transversely across the back of the trigonid, connecting the protoconid and metaconid. Mesial to the protocristid and running more or less parallel to it lies a transverse valley or groove. The M_2_ protoconid is especially reduced and raised only slightly above the talonid cusps. The protoconid is lower than the metaconid and paraconid and is relatively blunt, with a paracristid that slopes gradually from the apex, as in *I*. *graybullianus*. The postvallids of both M_1_ and M_2_ are canted mesially, resulting in very low-crowned teeth. Overall, the shape of the trigonid basin is rectangular. A weak buccal cingulid is present on M_1_, beginning at the base of the protoconid and terminating on the distal aspect of the hypoconid. The buccal cingulid is absent on M_2_. The cristid obliqua of M_1_ joins the postvallid slightly lingual to the protoconid, yielding a modest hypoflexid. On M_2_ the cristid obliqua joins the postvallid farther buccally, and the hypoflexid is very shallow as a result. The talonid is broad, shallowly excavated and heavily crenulated. The M_1_ hypoconid and entoconid are approximately equal in height, while the M_2_ entoconid is taller than the hypoconid.

M_3_ is notably low-crowned, partly because the mesial inclination of the trigonid is pronounced, so that the trigonid and talonid of M_3_ differ little in terms of height (Fig 4T–4V). As in other species of *Ignacius*, the trigonid of M_3_ is mesiodistally compressed, with weakly cuspidate protoconid, indistinct paraconid, and protocristid and paracristid that are barely raised to form weak borders around the trigonid. The talonid is extremely shallow, forming a relatively flat surface with heavy enamel crenulation on which the hypoconid and entoconid are nearly indiscernible from adjacent enamel crenulations and become incorporated within a raised cingulid that surrounds the periphery of the talonid.

### Phylogenetic analysis

**Fig 5:**
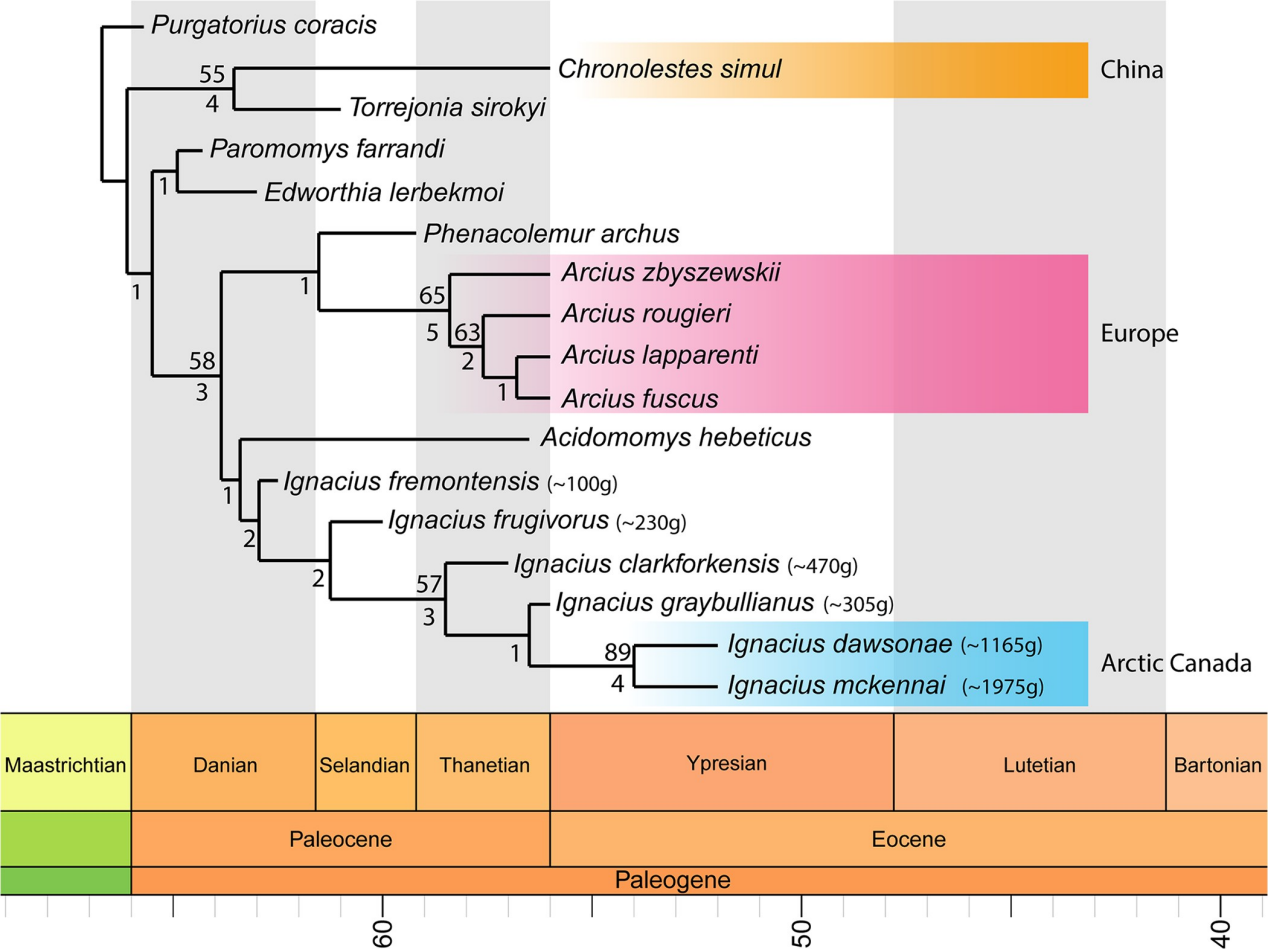
Results of maximum parsimony phylogenetic analysis depicted as a time-scaled phylogeny. Our analysis reconstructs the two new species of Arctic paromomyids as sister taxa that are deeply nested within the *Ignacius* clade. Heuristic searches yielded a single most parsimonious tree with 183 steps. Bootstrap support was calculated using 1000 replicates with replacement; absolute frequencies >50% are displayed above nodes. Bremer support values are displayed below nodes. Taxa not in color-coded boxes are geographically restricted to mid-latitudes of North America. Body size estimates for species of *Ignacius* based on M_1_ measurements follow Gingerich et al. [57]. Time-scaled phylogeny was generated using the R package strap “Stratigraphic Tree Analysis for Palaeontology” (see Materials and Methods). Note that estimated divergence dates are a product of the branch-scaling algorithm and do not accurately reflect documented biostratigraphic occurrences.

Analysis of our character-taxon matrix (S1 File) recovered a single most parsimonious tree characterized by strong congruence between the phylogenetic position and stratigraphic occurrence of individual species of *Ignacius* (Fig 5). In particular, the two new species from Ellesmere Island, which are the youngest well-documented species of *Ignacius* [56], are reconstructed as sister taxa. Indeed, the *I*. *mckennai* + *I*. *dawsonae* clade that is endemic to Ellesmere is among the most stable nodes on our tree, being supported in 89% of our bootstrap replicates. Species of *Ignacius* from mid-latitudes of North America comprise a pectinate series of older and increasingly distantly related lineages, with *I*. *graybullianus*, *I*. *clarkforkensis*, *I*. *frugivorus* and *I*. *fremontensis* forming successive outgroups to the Ellesmere clade.

### Dental topography

As noted in our qualitative descriptions, both species of *Ignacius* from Ellesmere Island have low-crowned molars, and these features can be quantified on the basis of relief index (RFI). M_1-2_ of *Ignacius mckennai* and *I*. *dawsonae* from Ellesmere show lower RFI values (0.4–0.44) than paromomyids from mid-latitudes (0.46–0.53) (Table 3; S1 Fig). The results of our ANOVA comparing M_1-2_ RFI values among paromomyid species came out highly significant (p <0.001). After performing Tukey’s HSD test on RFI, the only significant pairwise tests of M_1-2_ RFI were those tests including one Arctic and one mid-latitude species. Every test including *I*. *dawsonae* (with the exception of the test between *I*. *dawsonae* and *I*. *mckennai)* resulted in a significant p-value. Pairwise tests between *Ignacius mckennai* and *Phenacolemur pagei* and *I*. *clarkforkensis* were the only tests involving *I*. *mckennai* that yielded significant results. Results from the Tukey’s HSD tests can be found in S4 Table. The t-test comparing Arctic to mid-latitude paromomyid RFI values yielded a highly significant result (p < 0.001). Although M_3_ remains unknown for *I*. *mckennai*, the RFI values for M_3_ likewise show a significant difference (p = 0.041) between *I*. *dawsonae* from Ellesmere and mid-latitude taxa (Table 4). The significant difference in RFI between the Arctic *Ignacius* clade and other paromomyids is underscored by the absence of statistically significant differences in RFI values between *Ignacius* and other paromomyids (p = 0.179).

**Table 3:** Dental topography metrics for paromomyid lower molars.

Specimen	Species	Location	Locus	RFI	OPCR	ariaDNE	ln molar length
CMN 30986	*I*. *mckennai*	Arctic	M_1_	0.422	95.000	0.066	1.449
CMN 30850	*I*. *mckennai*	Arctic	M_2_	0.448	99.120	0.062	1.543
CMN 30828	*I*. *dawsonae*	Arctic	M_1_	0.426	74.750	0.064	1.289
CMN 30954	*I*. *dawsonae*	Arctic	M_1_	0.421	59.750	0.062	1.113
CMN 30999	*I*. *dawsonae*	Arctic	M_1_	0.426	106.000	0.068	1.266
CMN 30856	*I*. *dawsonae*	Arctic	M_2_	0.402	66.750	0.059	1.075
CMN 30889	*I*. *dawsonae*	Arctic	M_3_	0.353	185.750	0.065	1.611
CMN 30997	*I*. *dawsonae*	Arctic	M_3_	0.411	232.120	0.065	1.637
UM 108210	*I*. *clarkforkensis*	Mid-Lat	M_1_	0.527	61.000	0.056	0.846
UM 108210	*I*. *clarkforkensis*	Mid-Lat	M_2_	0.533	73.120	0.052	0.846
UM 108210	*I*. *clarkforkensis*	Mid-Lat	M_3_	0.465	90.000	0.046	1.206
AMNH 88309*	*I*. *fremontensis*	Mid-Lat	M_1_	0.500	98.880	0.064	0.542
AMNH 88309*	*I*. *fremontensis*	Mid-Lat	M_2_	0.482	94.880	0.068	0.536
UM 77268*	*I*. *frugivorus*	Mid-Lat	M_1_	0.464	77.620	0.066	0.610
UM 77268*	*I*. *frugivorus*	Mid-Lat	M_2_	0.504	94.750	0.064	0.678
USNM 493883	*I*. *graybullianus*	Mid-Lat	M_2_	0.512	80.250	0.069	0.793
USNM 9542*	*Pa*. *maturus*	Mid-Lat	M_1_	0.509	95.120	0.079	0.990
USNM 9542*	*Pa*. *maturus*	Mid-Lat	M_2_	0.466	103.380	0.072	1.037
USNM 9542*	*Pa*. *maturus*	Mid-Lat	M_3_	0.452	119.620	0.079	1.284
YPM-PU 14030*	*Ph*. *pagei*	Mid-Lat	M_1_	0.519	100.000	0.075	0.765
YPM-PU 14030*	*Ph*. *pagei*	Mid-Lat	M_2_	0.508	104.250	0.071	0.723
YPM-PU 14030*	*Ph*. *pagei*	Mid-Lat	M_3_	0.458	133.250	0.079	1.089

Specimens denoted by an asterisk indicate that scans were made from high-resolution epoxy casts. **Abbreviations**: Mid-Lat, Mid-Latitude.

**Table 4:** Results (p-values) of t-tests comparing dental topography metrics of paromomyid molars.

Data Partition	RFI	OPCR	ariaDNE
Arctic vs. midlatitude paromomyids (M_1-2_)	<0.001 (0.424/0.502)	0.482 (83.561/89.386)	0.325 (0.063/0.067)
Arctic vs. midlatitude paromomyids (M_3_)	0.041 (0.382/0.458)	0.029 (208.935/114.29)	0.850 (0.065/0.068)
*Ignacius* vs. all other paromomyids (M_1-2_)	0.179 (0.467/0.5)	0.048 (83.221/100.688)	0.001 (0.063/0.074)
Arctic *Ignacius* vs. midlatitude *Ignacius* (M_1-2_)	<0.001 (0.424/0.503)	0.946 (83.562/82.929)	0.792 (0.063/0.063)

Top numbers are p-values for each test, bottom numbers in parentheses are the means for each group being compared.

OPCR values were highly variable across M_1-2_ of paromomyids and sometimes even within individual species (Table 3; S1 Fig). For example, within the sample of *I*. *dawsonae*, OPCR values varied from 60–106, spanning the full range of OPCR values for the paromomyid sample as a whole (S1 Fig). *Paromomys* and *Phenacolemur* had OPCR values in the low 100s, which were the highest values among paromomyids aside from one specimen of *I*. *dawsonae*. The ANOVA comparing OPCR values among paromomyid species produced a non-significant result. However, M_1-2_ OPCR values for the entire sample of *Ignacius* differed significantly (p = 0.048) from those of other paromomyids (Table 4). Our M_3_ sample was small, comprising only two specimens of *I*. *dawsonae* and one specimen each of *I*. *clarkforkensis*, *Paromomys maturus*, and *Phenacolemur pagei* (Table 3). Despite the small sample size, M_3_ of *Ignacius dawsonae* yielded significantly higher (p = 0.029) OPCR values than those of mid-latitude paromomyids (Table 4).

All species of *Ignacius* produced relatively similar ariaDNE values, generally ~ 0.06. *Paromomys* and *Phenacolemur* yielded the highest ariaDNE values, in the 0.07 range (Table 3; S1 Fig). The ANOVA comparing ariaDNE values among paromomyid species produced a significant result (p <0.01). Only three of the pair-wise comparisons from the Tukey’s HSD tests of ariaDNE returned significant results. These were the tests between *Pa*. *maturus* and *I*. *clarkforkensis*, *Ph*. *pagei* and *I*. *clarkforkensis*, and *Pa*. *maturus* and *I*. *dawsonae*. There were no significant differences in ariaDNE values between Arctic and mid-latitude paromomyids when looking at either M_1-2_ data (p = 0.325) or M_3_ data (p = 0.85). To investigate the higher values seen in M_1-2_ of *Phenacolemur* and *Paromomys* further, we performed another test comparing species of *Ignacius* to other paromomyids. This test yielded a highly significant result (p = 0.001) (Table 4).

### Principal component analysis

**Fig 6:**
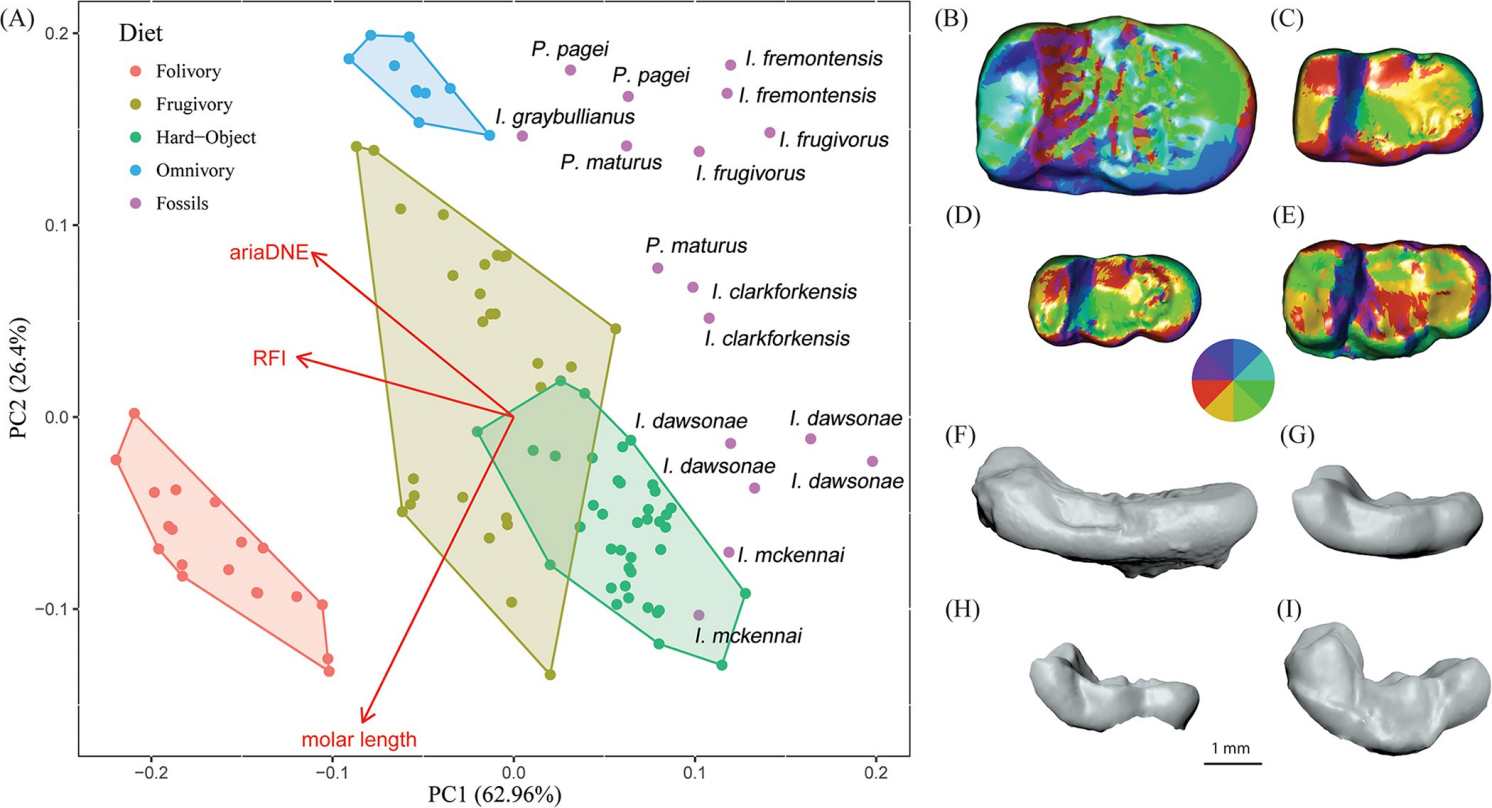
Results of principal component analysis and comparisons of M_3_ OPCR and crown height. (**A**) Principal component analysis showing dietary categories of extant platyrrhine primates and their relationship to fossil paromomyids. Shaded polygons represent the extant data points and their respective, assigned dietary categories. Fossil data are represented by purple circles. (**B-E**) Comparison of OPCR maps on lower third molars of four paromomyid species showing the increased complexity due to high enamel crenulation in *Ignacius dawsonae* **(B)**. (**F-I**) Buccal views of the corresponding teeth to show differences in crown height among paromomyids. (**B, F**) *Ignacius dawsonae* (CMN 30889); (**C, G**) *Ignacius clarkforkensis* (UM 108210); (**D, H**) *Phenacolemur pagei* (YPM-PU 14030); (**E, I**) *Paromomys maturus* (USNM 9542).

The results of our PCA using dental topography variables show that PC1 explains 63% of the variance while PC2 explains 26% and PC3 explains the remaining 11%. RFI and ariaDNE explain most of the variance along PC1 and molar length explains most of the variance along PC2 (Fig 6A). While there is some overlap between extant hard-object feeders and frugivores, extant folivores and omnivores occupy regions of morphospace that are distinct from other dietary categories (Fig 6A). In general, the fossil paromomyid taxa plot higher along PC1 than the extant platyrrhine sample, reflecting the fact that dental topography metrics for paromomyids differ systematically from those of the extant platyrrhine sample. Within the paromomyid sample, three clusters are distributed primarily across PC2. The two Arctic taxa (*I*. *mckennai* and *I*. *dawsonae*) cluster within or near the hard-object feeding platyrrhines (lower on PC2), while many of the more basal *Ignacius* species, along with *Paromomys* and *Phenacolemur*, cluster higher along PC2, closer to the omnivorous platyrrhines. *Ignacius clarkforkensis* and one specimen of *Pa*. *maturus* occupy an intermediate position with respect to these two clusters along PC2, closest to the frugivorous platyrrhines.

The results from our MANOVA show a highly significant (p<0.001) difference among dietary groups. To further understand the differences in diet of our sample, we performed three ANOVAs comparing diet along each principal component. All three of these ANOVAs produced highly significant results (p<0.001) and so these were followed up by Tukey’s HSD tests (S5 Table). The only non-significant test (p = 0.22) of diet along PC1 was the comparison between extant hard object feeders and mid-latitude paromomyids. Diet comparisons of PC2 values produced mainly significant results, although the comparisons of Arctic paromomyids versus folivores (p = 0.84), Arctic paromomyids versus hard-object feeders (p = 0.98), and hard-object feeders versus folivores (p = 0.94) were non-significant. Approximately half of the pairwise diet comparisons of PC3 values were non-significant. The full results can be found in S2 Fig and S5 Table.

While there is overlap in lower molar length (a proxy for body mass) between the extant platyrrhine sample and fossil paromomyids, the paromomyids tend to be smaller (S3 File). To investigate the potentially confounding effects of these systematic differences in lower molar length on our PCA, we constructed a bivariate plot of ariaDNE versus RFI for the combined sample of extant platyrrhines and fossil paromomyids (S3 Fig). On the basis of these dental topography metrics alone, dietary categories of extant platyrrhines occupy distinct regions of morphospace, although there is broad overlap among groups. Extant platyrrhine hard-object feeders are distinguished from other platyrrhines by having low values of both ariaDNE and RFI. Arctic paromomyids cluster nearest the extant hard-object feeders in S3 Fig, although none of the Arctic paromomyids occur within the extant hard-object feeder morphospace. There is complete overlap in ariaDNE values between Arctic paromomyids and extant platyrrhine hard-object feeders, but the Arctic paromomyids uniformly show lower RFI values. The mid-latitude sample of paromomyids is distibuted within or near the morphospace occupied by extant platyrrhine frugivores and hard-object feeders (S3 Fig).

## Discussion

### Phylogeny and historical biogeography of Arctic paromomyids

**Fig 7:**
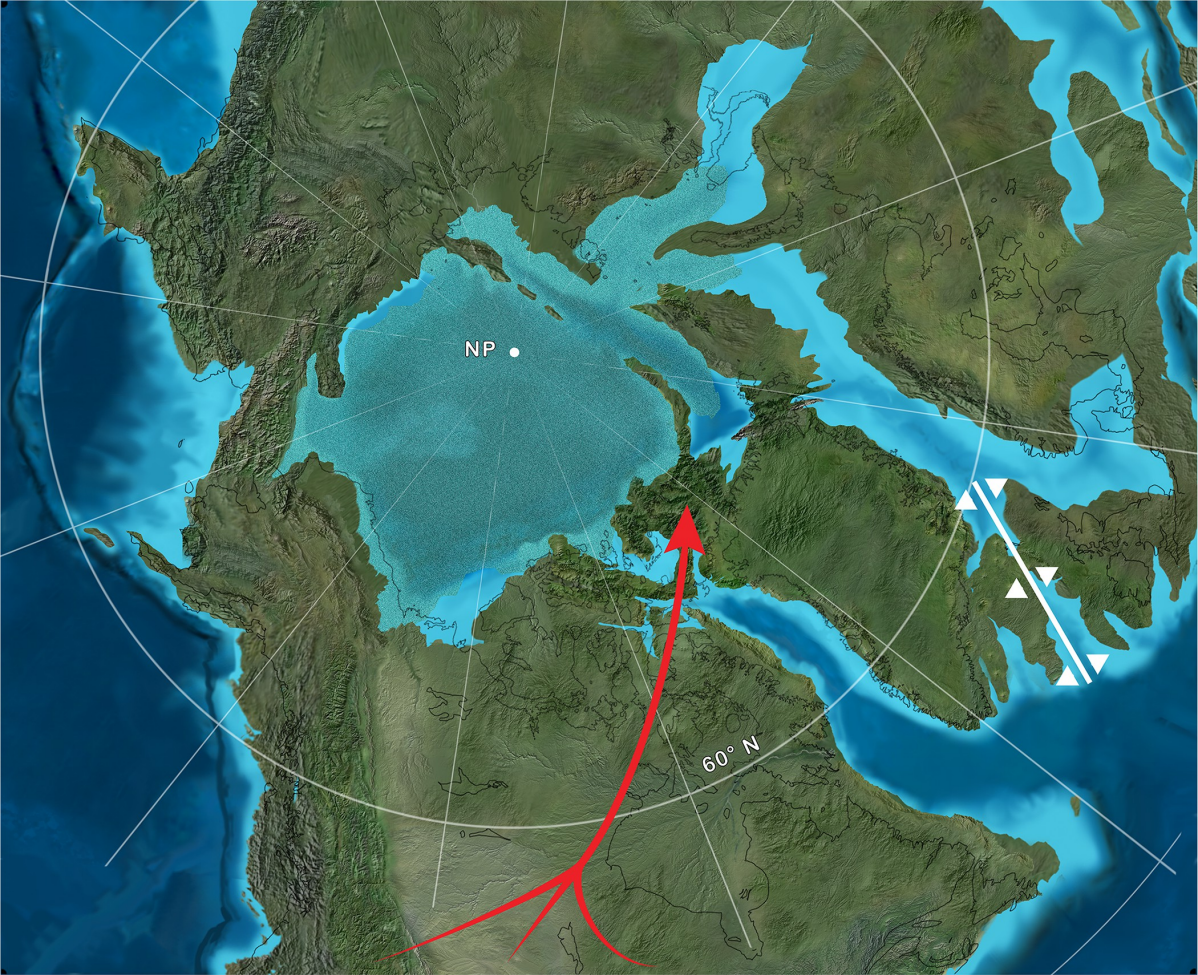
Paleogeographic map of northern Laurasia during the EECO. Red arrow with multiple tails indicates continuity of terrestrial dispersal routes from mid-latitude regions of North America to Ellesmere Island in the late early Eocene. The relative stability of these terrestrial dispersal routes through time facilitated colonization of Ellesmere by multiple North American mammal clades, including *Ignacius*, *Anacodon*, Plagiomenidae and Brontotheriidae. In contrast, rifting in the North Atlantic Igneous Province [58], designated by the white graphic between Greenland and what is now Ireland/United Kingdom, had apparently proceeded sufficiently by the late early Eocene as to preempt routine dispersal by terrestrial mammals between western Europe and Ellesmere Island. NP, paleopole position of North Pole. Source map © 2019, Paleogeography of the Arctic, Colorado Plateau Geosystems Inc.

Our phylogenetic results indicate that *Ignacius mckennai* and *I*. *dawsonae* from the late early Eocene of Ellesmere Island are sister taxa, comprising a clade with multiple successive outgroups from older sites across mid-latitude regions of North America (Fig 5). Phylogenetic data therefore support a single (monophyletic) colonization of Arctic Canada by *Ignacius*, followed by in situ diversification to yield the sister taxa *I*. *mckennai* and *I*. *dawsonae*. Furthermore, because multiple outgroups for the Arctic *Ignacius* clade are known from mid-latitude regions of North America, we infer that *Ignacius* colonized Ellesmere from lower latitudes of North America rather than Europe, where only more distantly related paromomyids are known [27]. A similar pattern of colonization of Ellesmere by North American, rather than European, mammals is indicated by the Ellesmere occurrences of *Anacodon* (Arctocyonidae), plagiomenids and brontotheres, none of which is known from the early Eocene of Europe [6, 8, 11]. A plausible explanation for the prevalence of North American mammal clades in the Ellesmere fauna is that rifting in the North Atlantic Igneous Province had progressed sufficiently by the late early Eocene that it severely restricted European clades from colonizing Arctic Canada (Fig 7) [58].

The highly nested phylogenetic position of the Ellesmere *Ignacius* clade helps constrain when these animals colonized Arctic Canada. The sister group of the Ellesmere *Ignacius* clade, *I*. *graybullianus*, is documented from the early Eocene (Wa1-Wa4) of the Bighorn Basin in Wyoming [59]. At mid-latitudes, *I*. *graybullianus* is locally extirpated just before the onset of a warming trend that anticipates the beginning of the EECO [60]. The persistence of the Ellesmere clade of *Ignacius* into the late early Eocene suggests that the warmer conditions of the EECO enabled *Ignacius* to colonize high-latitude regions of Arctic Canada and subsequently diversify there (Fig 7). However, the stratigraphic range and phylogenetic position of the European paromomyid *Arcius* suggest an earlier episode of paromomyid dispersal across high latitude regions of North America that may have included Ellesmere. Species of *Arcius* are documented from very early Eocene sites such as Silveirinha in Portugal and Sotteville-sur-Mer in Normandy [27], but *Arcius* is rooted more deeply on the paromomyid tree, having a potential sister group in the late Paleocene (Tiffanian) North American species *Phenacolemur archus* (Fig 5). These data are consistent with *Arcius* having dispersed across high latitude regions of North America during the earlier Paleocene-Eocene Thermal Maximum (PETM) hyperthermal event. If so, *Arcius* would likely have been a transient resident of Ellesmere and its vicinity, because the PETM lasted only ~200 Ka. The PETM stratigraphic interval has recently been recognized at Stenkul Fiord on southern Ellesmere Island, although the local section spanning the PETM is currently devoid of fossil vertebrates [61].

### Dental and gnathic adaptations of Arctic paromomyids

The Ellesmere *Ignacius* clade is highly autapomorphous with respect to other paromomyids, as might be expected of the only Paleogene primatomorphan to succeed in colonizing the high Arctic during the extreme environmental conditions of the EECO. Unfortunately, dental and gnathic features are all that is currently known of the anatomy of Ellesmere paromomyids, so we cannot assess whether further modifications of the cranial or appendicular skeleton contributed to their adaptive success. Closely related paromomyids that are documented by postcranial features show clear evidence of being committed arborealists [24, 28, 42], and there is no reason to assume that the Ellesmere *Ignacius* clade was any less arboreal than paromomyids from mid-latitudes. Given the limited nature of our knowledge of the anatomical modifications characterizing the Ellesmere *Ignacius* clade, does any coherent picture emerge about their paleoecological or dietary adaptations?

Evidence for a rostral shift in the origin of the superficial masseter (based on the relatively rostral position of the zygomatic arch in *I*. *mckennai*) and a rostral shift in the insertion of the masseter and the zygomatic and superficial temporalis (based on the forward extension of the masseteric fossa on the dentary in *I*. *dawsonae*) implies elevated bite forces at multiple cheek tooth loci in the Ellesmere *Ignacius* clade relative to paromomyids from mid-latitude regions of North America and Europe (Fig 3) [62, 63]. In these features, the Ellesmere *Ignacius* clade is partly convergent upon the highly autapomorphous plesiadapid *Chiromyoides*, which shows dental and gnathic adaptations for extractive foraging akin to that of the extant lemur *Daubentonia* and the extant phalangeroid marsupial *Dactylopsila* [62, 64]. In contrast to *Chiromyoides* and *Daubentonia*, there is no evidence for a reduction in molar occlusal area in the Ellesmere *Ignacius* clade, implying that a dietary focus on high quality foods requiring little mastication (like insect larvae) was absent in Ellesmere species of *Ignacius*.

In general, the results of our dental topographic analysis and principal component analysis are consistent with biomechanical evidence for elevated bite forces in Ellesmere species of *Ignacius*. For example, Arctic paromomyids (especially *I*. *dawsonae*) have significantly lower RFI values (and therefore lower-crowned molars) than their close relatives from mid-latitudes (Tables 3 and 4). Additionally, Arctic paromomyids show lower RFI values than extant platyrrhine hard-object feeders (S3 Fig). Low-crowned molars are generally associated with frugivory and hard-object feeding in extant primates [33]. Paromomyids in general are known to exhibit low-crowned molars (Fig 6F–6I) [65], so it is interesting that the Arctic taxa have further exaggerated this feature, resulting in molars that are relatively flat with little topographic relief aside from strong enamel crenulation. The extremely low molar crowns seen in Arctic paromomyids (Fig 6F; S3 Fig) are reminiscent of extant primates such as pitheciine platyrrhines that specialize on hard-object feeding. The low molar crowns, reduced cusps, and crenulated enamel of pitheciines yield a remarkably similar molar morphology to that shown by the Ellesmere *Ignacius* clade, suggesting that their teeth evolved convergently to perform a similar function.

Another dental topographic metric, OPCR, has historically been less effective at reconstructing diet in primates [65], although frugivores and hard-object feeders generally exhibit higher OPCR values [33]. OPCR is a quantification of tooth complexity, such that teeth with more cusps, crests, or enamel crenulation will produce higher OPCR scores. Development of enamel crenulation seems to vary according to tooth locus in the Ellesmere *Ignacius* clade, with M_3_ being particularly heavily crenulated (Fig 4T). Increased complexity in the form of heavy crenulation yields very high OPCR values for M_3_ in *I*. *dawsonae* from Ellesmere Island (Fig 6B; Table 3), which differs significantly from OPCR scores for M_3_ in paromomyids from mid-latitudes (Table 4). Enamel crenulation has often been associated with hard-object feeding, but this phenomenon is present in a wide range of mammals showing a variety of dietary specializations [66].

Our principal component analysis segregates the Ellesmere *Ignacius* clade from all other paromomyids (Fig 6A). *Ignacius mckennai* and *I*. *dawsonae* plot well below *I*. *frugivorus*, *I*. *fremontensis*, *I*. *graybullianus*, *Paromomys maturus* and *Phenacolemur pagei* along PC2, while *I*. *clarkforkensis* falls between these two clusters. Because most of the variance along PC2 is explained by lower molar length, the cluster of large Arctic paromomyids to the exclusion of the remaining taxa is not unexpected. Excluding lower molar length shows that Arctic paromomyids are distinguished from both mid-latitude paromomyids and extant platyrrhines in having lower RFI values (S3 Fig). The results of both the PCA and bivariate plot of dental topography metrics indicate that Arctic paromomyids are most closely aligned with extant platyrrhine hard-object feeders.

**Fig 8:**
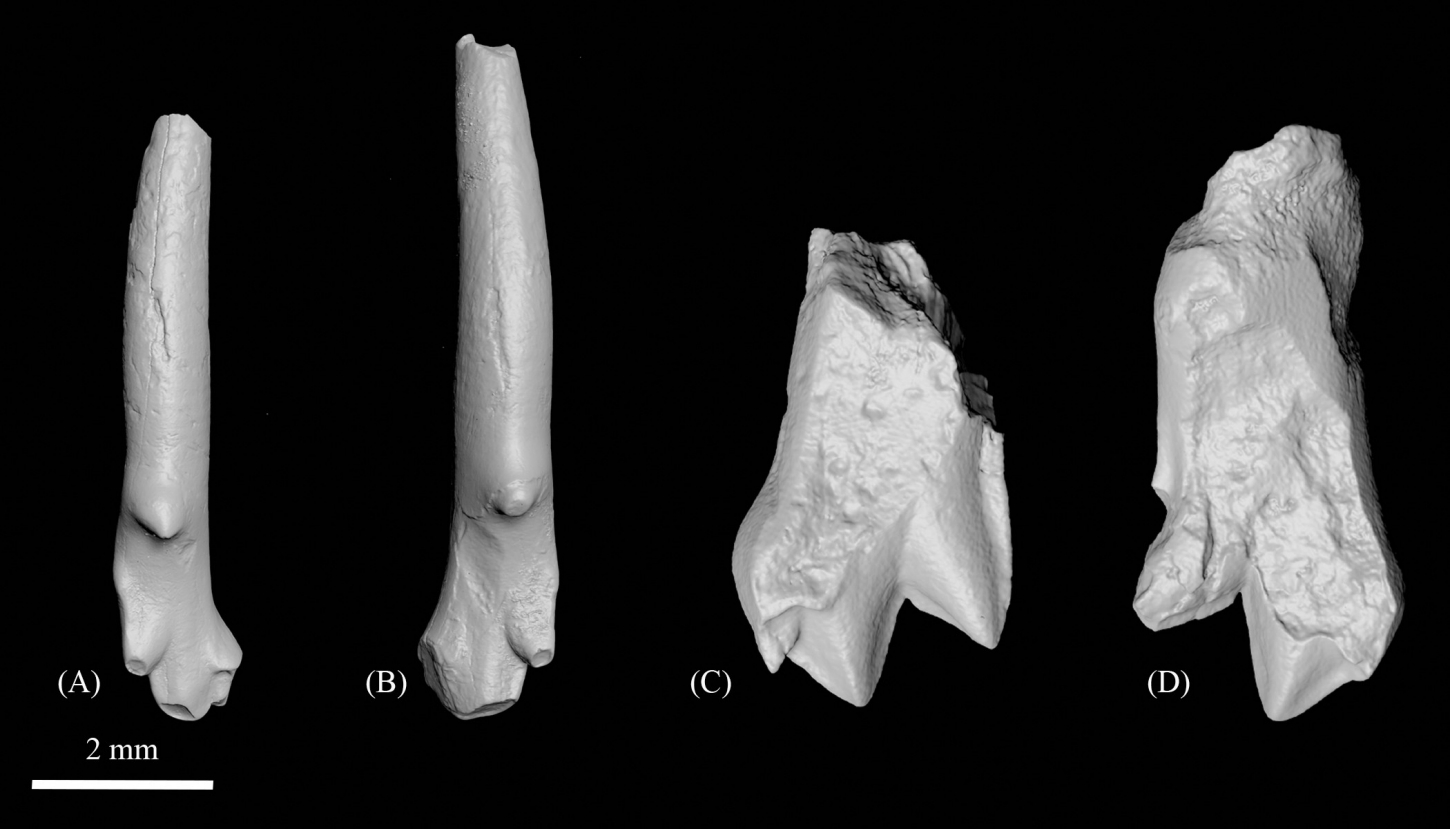
Comparison of I^1^ wear patterns in Arctic *Ignacius dawsonae* and mid-latitude *Ignacius frugivorus*. All specimens are depicted in lingual view. (**A**) Left I^1^ of *Ignacius frugivorus* (KUVP 156588); (**B**) Right I^1^ of *Ignacius frugivorus* (CM 73725); (**C**) Right apical fragment of I^1^ of *Ignacius dawsonae* (CMN 32325); (**D**) Left I^1^ of *Ignacius dawsonae* (CMN 30903).

The most surprising aspect of I^1^ morphology in the Ellesmere *Ignacius* clade is the heavy lingual, as opposed to apical, wear shown by both known incisor specimens of *I*. *dawsonae* (Fig 4A and 4C). This stands in contrast to I^1^ of *I*. *frugivorus*, which can show moderate apical wear on the anterocone and laterocone while the lingual surface of the crown remains relatively pristine (Fig 8).

Taken together, the dental and gnathic evidence pertaining to the Ellesmere *Ignacius* clade highlights the autapomorphous nature of these taxa in relation to paromomyids from mid-latitudes of North America, including earlier species of *Ignacius*. The unusual lingual wear on I^1^, the rostral shift in origin and insertion of masticatory muscles, and dental topographic features including heavy enamel crenulation and low RFI values are all consistent with an emphasis on hard-object feeding in Arctic species of *Ignacius*. Although the precise nature of the dietary adaptations of the Ellesmere *Ignacius* clade remains unclear, it seems obvious that these animals were forced to modify their diets in order to colonize the physiognomically distinct and environmentally challenging ecosystem of Ellesmere Island during the EECO [4]. One possibility that merits further evaluation is that seeds and nuts provided a fallback food resource that enabled *Ignacius mckennai* and *I*. *dawsonae* to survive long intervals of winter darkness, when fruits and other plant-based resources would have been in short supply. Intriguingly, other Eocene mammals from Ellesmere Island likewise show evidence of utilizing fallback foods in response to seasonal photoperiodicity. Isotopic data from tooth enamel of the pantodont *Coryphodon* indicate that these large terrestrial herbivores adopted alternative winter diets that may have included wood and leaf litter (rather than live leaves), evergreen conifers, and fungi [67]. Reliance on fallback foods during winter darkness may therefore have been a relatively common strategy among mammals inhabiting Ellesmere Island during the early Eocene.

### Delimiting the potential scope and inherent limitations of biotic change in a rapidly warming Arctic

The EECO represents the most intense and most prolonged interval of global warming of the Cenozoic [68]. The late early Eocene biota of the Margaret Formation on Ellesmere Island evolved under the hyperthermal conditions of the EECO, providing an ideal model for examining how an ancient Arctic ecosystem was transformed in the face of rapid global warming. The presence of thermophilic vertebrates including crocodilians, tapiroids and primatomorphans on Ellesmere during the EECO underscores the wide range of potentially invasive taxa that could colonize a significantly warmer Arctic in the future. However, even the hyperthermal conditions of the EECO did not enable wholesale colonization of the Arctic by thermophilic mammals. Instead, a high level of taxonomic selectivity is illustrated by the fact that a single clade of paromomyids colonized Ellesmere during the EECO, during an interval when primatomorphans were highly diverse and frequently abundant across mid-latitudes of North America. For example, the mammalian fauna of the Lost Cabin Member of the Wind River Formation in central Wyoming includes multiple species of adapiform and omomyid euprimates as well as paromomyid and microsyopid primatomorphans [69, 70]. In terms of relative abundance, euprimates and microsyopids were far more common than paromomyids in the late early Eocene of central Wyoming [69], yet these taxa apparently failed to colonize Ellesmere during the EECO, while the paromomyid *Ignacius* succeeded in doing so. The taxonomic selectivity exemplified by the primatomorphan colonization of Ellesmere Island contributed to an overall pattern of lower alpha diversity in the Arctic than at mid-latitudes during the Eocene [3], mirroring diversity patterns that prevail in the Arctic today [71].

In addition to a high level of taxonomic selectivity, significant morphological transformations were associated with the primatomorphan colonization of Ellesmere during the EECO. The evolutionary changes that were directly associated with colonization of the Arctic by *Ignacius* reflect a shift in feeding strategy, perhaps in response to the polar light regime and unique physiognomy of the Arctic flora [4]. Body size more than doubled from the largest mid-latitude species of *Ignacius* (*I*. *clarkforkensis*) to the smaller of the two Arctic species (*I*. *dawsonae*), while the larger Arctic species of *Ignacius* (*I*. *mckennai*) boasts a body mass that was more than quadruple that of any mid-latitude species of *Ignacius* (Fig 5). Significantly larger body size along with a reduction of molar crown height, development of elaborate enamel crenulations, and a rostral translation in the line of action of major muscles of mastication indicate a dietary shift toward hard objects, either as a primary or fallback food resource during long intervals of polar darkness. The relatively shallow latitudinal temperature gradient that characterized the EECO meant that Arctic photic seasonality was probably a more important factor than climate in screening which mammalian taxa were able to colonize Ellesmere during the EECO. Eberle et al. [67] have made a similar case with respect to the terrestrial ungulate fauna of Ellesmere.

Several of the mammal clades that colonized Ellesmere Island during the EECO, including the microparamyine rodent *Strathcona*, plagiomenids and *Ignacius*, experienced minor to moderate diversification there [6, 7]. Biogeographically, Arctic Canada during the EECO may have functioned more like a large island at lower latitude than the modern Arctic, inasmuch as colonization conferred a high capacity to instigate radiation. These radiations possibly reflect the imbalanced nature of the Ellesmere mammal fauna, which lacks multiple taxa that dominated contemporary mid-latitude ecosystems in North America [3].

Predictions of future biotic change in the Arctic based on species distribution models suggest an increase in species richness caused by the northward range expansion of multiple taxa as climate becomes progressively warmer [72]. Patterns of biotic change during the EECO on Ellesmere Island show that with sufficient warming, even such thermophilic mammals as primatomorphans can colonize high latitude regions. Under a very shallow latitudinal temperature gradient like that of the EECO, photic seasonality appears to pose the most significant barrier to Arctic colonization. As Arctic ecosystems are transformed by anthropogenic warming, multiple evolutionary factors including selective colonization, in situ diversification, and lineage-specific modification of ancestral morphologies will influence the nature of faunal dynamics at high northern latitudes.

## Supporting information

S1 Table Provenance of paromomyid specimens from the Margaret Formation, Ellesmere Island. (DOCX)

S2 Table List of characters and character states used in phylogenetic analysis. (DOCX)

S3 Table First occurrence data for taxa included in the phylogenetic analysis. (DOCX)

S4 Table Results of Tukey’s HSD tests for RFI and ariaDNE. (DOCX)

S5 Table Results of the Tukey HSD tests comparing principal components by diet. (DOCX)

S1 File Character-taxon matrix used in phylogenetic analysis. (TXT)

S2 File Dental topographic data for fossil paromomyid specimens used in the analysis. (CSV)

S3 File Dental topographic data for combined sample of extant platyrrhines and fossil paromomyids. (CSV)

S4 File PC scores for individual specimens used in the analysis. (CSV)

S5 File R script used for statistical analyses. (R)

S1 Fig Boxplot showing observed range and mean values for dental topography metrics among fossil paromomyids. (TIF)

S2 Fig Boxplot showing observed range and mean PC scores for extant dietary categories and fossil paromomyids (partitioned into Arctic and mid-latitude samples). (TIF)

S3 Fig Scatterplot showing the relationship between ariaDNE and RFI of Arctic and mid-latitude paromomyids compared to platyrrhine diet categories. (TIF)
