# Supplementary material for: Basal Primatomorpha colonized Ellesmere Island (Arctic Canada) during the hyperthermal conditions of the early Eocene climatic optimum
Source: PLoS One. 2023 Jan 25;18(1):e0280114. doi: 10.1371/journal.pone.0280114 (PMC9876366; doi:10.1371/journal.pone.0280114)
Supplement: S1 Table — (DOCX) [file pone.0280114.s001.docx]

**S1 Table. Provenance of paromomyid specimens from the Margaret Formation, Ellesmere Island.**

| Taxon | Specimen | Locality | Element | Morphosource doi |
| --- | --- | --- | --- | --- |
| *Ignacius mckennai* | CMN 30830 (holotype) | 76-85 | L maxilla M^1^ | 10.17602/M2/M439502 |
| *Ignacius mckennai* | CMN 30850 | 76-56 | L dentary M_2_ | 10.17602/M2/M439505 |
| *Ignacius mckennai* | CMN 30986 | 76-85 | L dentary M_1_ | 10.17602/M2/M439508 |
| *Ignacius dawsonae* | CMN 30828 | 76-85 | R M_1_ | 10.17602/M2/M439511 |
| *Ignacius dawsonae* | CMN 30831 | 76-85 | L dentary M_3_ |  |
| *Ignacius dawsonae* | CMN 30835 | 76-44 | L edentulous dentary |  |
| *Ignacius dawsonae* | CMN 30837 (holotype) | 76-85 | R dentary P_4_-M_1_ | 10.17602/M2/M439514 |
| *Ignacius dawsonae* | CMN 30853 | 76-44 | R edentulous dentary |  |
| *Ignacius dawsonae* | CMN 30856 | 76-49 | R M_2_ | 10.17602/M2/M439517 |
| *Ignacius dawsonae* | CMN 30864 | 76-84 | L M_2_ |  |
| *Ignacius dawsonae* | CMN 30867 | 76-85 | L M_2_ |  |
| *Ignacius dawsonae* | CMN 30868 | 76-85 | L M^2^ | 10.17602/M2/M439520 |
| *Ignacius dawsonae* | CMN 30883 | 76-85 | L M_3_ | 10.17602/M2/M439523 |
| *Ignacius dawsonae* | CMN 30889 | 76-85 | L M_3_ | 10.17602/M2/M439526 |
| *Ignacius dawsonae* | CMN 30902 | 76-85 | R M_3_ | 10.17602/M2/M439530 |
| *Ignacius dawsonae* | CMN 30903 | 76-85 | L I^1^ | 10.17602/M2/M439533 |
| *Ignacius dawsonae* | CMN 30927 | 76-85 | R M_3_ talonid |  |
| *Ignacius dawsonae* | CMN 30933 | 76-85 | L M_3_ |  |
| *Ignacius dawsonae* | CMN 30936 | 76-85 | L P_4_ | 10.17602/M2/M439536 |
| *Ignacius dawsonae* | CMN 30949 | 76-44 | L P_4_ | 10.17602/M2/M439539 |
| *Ignacius dawsonae* | CMN 30954 | 76-44 | L M_1_ | 10.17602/M2/M439542 |
| *Ignacius dawsonae* | CMN 30959 | 76-44 | R M_1_ | 10.17602/M2/M439545 |
| *Ignacius dawsonae* | CMN 30988 | 76-85 | L M_3_ talonid | 10.17602/M2/M439585 |
| *Ignacius dawsonae* | CMN 30995 | 77-85 | R M^1^ | 10.17602/M2/M439548 |
| *Ignacius dawsonae* | CMN 30996 | 77-85 | R M^2^ | 10.17602/M2/M439551 |
| *Ignacius dawsonae* | CMN 30997 | 77-85 | L M_3_ | 10.17602/M2/M439554 |
| *Ignacius dawsonae* | CMN 30998 | 77-85 | R M^2^ | 10.17602/M2/M439557 |
| *Ignacius dawsonae* | CMN 30999 | 77-85 | L M_1_ | 10.17602/M2/M439560 |
| *Ignacius dawsonae* | CMN 32320 | 77-85 | R M^1^ | 10.17602/M2/M439563 |
| *Ignacius dawsonae* | CMN 32321 | 77-85 | L M^3^ | 10.17602/M2/M439566 |
| *Ignacius dawsonae* | CMN 32325 | 76-85 | R I^1^ | 10.17602/M2/M439576 |
