# Supplementary material for: Basal Primatomorpha colonized Ellesmere Island (Arctic Canada) during the hyperthermal conditions of the early Eocene climatic optimum
Source: PLoS One. 2023 Jan 25;18(1):e0280114. doi: 10.1371/journal.pone.0280114 (PMC9876366; doi:10.1371/journal.pone.0280114)
Supplement: S2 Table — (DOCX) [file pone.0280114.s002.docx]

**S2 Table. List of characters and character states used in phylogenetic analysis.**

Characters highlighted in blue are additions to the matrix developed by López-Torres and Silcox (2018) [27].

**General Characters**

1. Enamel crenulation in upper/lower molar basins (ordered)

0: Absent

1: Moderate crenulation in some/all molars

2: Strong crenulation in all molar basins

**Upper incisors**

1. Presence of posterocone on I1

0: Absent

1: Present

1. Relative height anterocone/mediocone on I1

0: Anterocone taller than mediocone

1: Mediocone taller than anterocone

1. Position of the laterocone in relation to the anterocone

0: Laterocone positioned at the base of anterocone

1: Laterocone approaching the height of the anterocone

**Upper premolars**

1. Presence of P2

0: Absent

1: Present

1. Presence of metacone on P4

0: Absent

1: Present

1. Presence of a molariform P4

0: P4 with a metacone signiﬁcantly smaller than the paracone and no expanded distolingual basin

1: P4 with a metacone approaching in size to the paracone and an expanded distolingual basin

1. Presence of precingulum on P4

0: Absent

1: Present

1. Presence of parastyle on P4

0: Absent

1: Present

1. Shape of P4 (ordered)

0: T-shaped

1: Triangular

2: Quadrangular

1. Mesial parastylar expansion on P4

0: Projecting beyond the mesial border

1: Not projecting

1. Acuteness of P4 cusps

0: Acute

1: Bulbous

1. Height of postprotocingulum on P4

0: Low (crest dips closer to the roots)

1: High (crest stays near the tip of the protocone in height)

**Upper molars**

1. Position of zygomatic arch in relation to M1

0: Root of zygomatic begins distal to the mesial border of M1

1: Root of zygomatic begins at mesial edge of M1

1. Depth of distolingual basin on M1-2

0: Shallow

1: Deep

1. Presence of conules on M1-2 (ordered)

0: Both conules present

1: Metaconules absent

2: Both conules absent

1. Parastylar expansion on M1-2

0: No expansion

1: Expanded

1. Outline of M1

0: Squared

1: Rectangular and narrow

1. Shape of the ectoflexus (buccal margin) on M1-2

0: invaginated ectoflexus

1: non-invaginated ectoflexus

1. Slope of postprotocingulum

0: steep slope

1: gradual slope

1. Presence of a preprotocingulum

0: absent

1: present

1. Morphology of the precingulum and mesiobuccal cingulum on M^1-2^ (ordered)

0: No precingulum

1: Short precingulum (precingulum runs buccally from the base of the protocone but ends at or before paraconule), no overlap with the mesiobuccal cingulum

2: long precingulum (precingulum runs buccally from the base of the protocone past the paraconule), no overlap with the mesiobuccal cingulum

3: long precingulum that overlaps the mesiobuccal cingulum creating a “stair step” morphology

1. Depth of trigon basin on M1-2

0: Shallow

1: Deep

1. Presence of postmetaconule crista on M1-2

0: Absent

1: Present

1. V shaped postparacrista/premetacrista on M1

0: Absent

1: Present

1. Height of metacone in relation to height of the paracone of M1

0: Paracone and metacone equal in heigh

1: Metacone significantly lower than paracone

1. Expansion of mesiolabial corner on M3

0: Not expanded, buccal border is straight

1: Expanded

1. Expansion of distolingual basin on M3 (ordered)

0: No expansion (distolingual basin does not expand beyond metacone)

1: Slightly expanded (distolingual basin expands slightly beyond metacone)

2: Significantly expanded (distolingual basin becomes a hypocone lobe)

**Lower canine**

1. Presence of C1

0: Present

1: Absent

**Lower premolars**

1. Presence of P2

0: Present

1: Absent

1. Presence of P3

0: Present

1: Absent

1. Trigonid/talonid width proportion on P4

0: Talonid as wide as or wider than trigonid

1: Talonid narrower than trigonid

1. P4/M1 width proportion

0: P4 narrower than M1

1: P4 of approximately the same width as M1

1. Width at the base of the P4 protoconid

0: Narrowly based protoconid

1: Broadly based protoconid

1. Presence of a mesial bulge in the base of the P4 protoconid

0: Absent

1: Present

1. P4/M1 area proportion

0: Small P4 area compared to M1 area

1: Similar

1. Relative mesiodistal length of P4 to M1

0: P4 shorter than M1

1: P4 equal or longer than M1

1. Morphology of the hypoﬂexid

0: Distinct, deep

1: Not distinct, shallow

1. Presence of paracristid

0: Present

1: Absent

1. Relative length of the talonid compared to the length of the tooth

0: Relatively short talonid (less than 26% of the tooth length)

1: Relatively long talonid (more than 26% of the tooth length)

1. Presence of a crest connecting the protoconid and the hypoﬂexid fold (prehypoﬂexid cristid)

0: Absent

1: Present

1. Presence of a metaconid

0: Absent

1: Lingual expansion of the protoconid (not yet a full metaconid)

2: Present

**Lower molars**

1. Length of trigonid

0: Trigonids become less mesiodistally compressed from M1 to M3, or there is no change

1: Trigonids become more mesiodistally compressed from M1 to M3

1. Shape of the protocristid on M1

0: V-shaped

1: Slightly concave

1. Presence of distal cingulid on M1 and M2

0: Absent

1: Present

1. Presence of hypoconulid on M1 and M2

0: Absent

1: Present

1. Presence of buccal cingulid on M1 and M2 trigonids

0: Absent

1: Present

1. Presence of buccal cingulid on M1 and M2 talonids

0: Absent

1: Present

1. Shape of the M1 trigonid basin

0: Semicircular

1: Squared

2: Triangular

1. Mesial inﬂection of the M1 and M2 trigonids (ordered)

0: Absent/weak

1: Somewhat pronounced

2: Very pronounced

1. Relative height of the hypoconid compared to the entoconid on M1

0: Hypoconid taller than entoconid

1: Subequal

2: Entoconid taller than hypoconid

1. Relative height of the protoconid compared to the metaconid on M1

0: Protoconid taller than metaconid

1: Subequal

2: Metaconid taller than protoconid

1. Presence of paraconid on M2

0: Absent

1: Present

1. Distinctiveness of the M2 paraconid relative to the M1 paraconid

0: Comparably distinct to the M1 paraconid

1: Less distinct than the M1 paraconid

1. Relative height of the paraconid compared to the metaconid on M2

0: Paraconid lower than metaconid

1: Paraconid subequal of taller than metaconid

1. Relative height of the hypoconid compared to the entoconid on M2

0: Hypoconid taller than entoconid

1: Subequal

2: Entoconid taller than hypoconid

1. Relative height of the protoconid compared to the metaconid on M2

0: Protoconid taller than metaconid

1: Subequal

2: Metaconid taller than protoconid

1. Acuteness of cusps

0: Relatively acute

1: Blunter

1. Presence of M3 paraconid

0: Absent

1: Present

1. Relative height of the hypoconid compared to the entoconid on M3

0: Hypoconid taller than entoconid

1: Subequal

2: Entoconid taller than hypoconid

1. Relative height of the protoconid compared to the metaconid on M3

0: Protoconid taller than metaconid

1: Subequal

2: Metaconid taller than protoconid

1. M3 trigonid basin area

0: Small basin, straight at the front

1: Expansive trigonid basin, curved at the front

1. Morphology of the M3 hypoconulid lobe

0: From a distal view, the central occlusal surface is taller than the sides

1: from a distal view, the medial and later edges are taller than the central occlusal surface
