# Supplementary material for: Basal Primatomorpha colonized Ellesmere Island (Arctic Canada) during the hyperthermal conditions of the early Eocene climatic optimum
Source: PLoS One. 2023 Jan 25;18(1):e0280114. doi: 10.1371/journal.pone.0280114 (PMC9876366; doi:10.1371/journal.pone.0280114)
Supplement: S3 Table — (DOCX) [file pone.0280114.s003.docx]

**S3 Table. First occurrence data for taxa included in the phylogenetic analysis.**

| Taxon | First occurrence | Reference | Biostratigraphy | Geochronology |
| --- | --- | --- | --- | --- |
| *Purgatorius coracis* | Rav W-1 level, Medicine Hat Brick and Tile Quarry, Saskatchewan | Fox & Scott (2011) | Pu-2 | 65.7 Ma |
| *Chronolestes simul* | Wutu local fauna, Shandong Province, PRC | Beard & Wang (1995) | Bumbanian | 56 Ma |
| *Torrejonia sirokyi* | Saddle Locality, Bison Basin, WY | Szalay (1973) | Ti-2 | 61 Ma |
| *Paromomys farrandi* | Farrand Channel local fauna, Williston Basin, MT | Clemens & Wilson (2009) | To-1 | 64.3 Ma |
| *Edworthia lerbekmoi* | Edworthy Park locality, Calgary, Alberta | Fox et al. (2010) | To-2 | 63 Ma |
| *Phenacolemur archus* | Croc Tooth Quarry, Bighorn Basin, WY | Secord (2008) | Ti-4b | 59.2 Ma |
| *Arcius zbyszewskii* | Silveirinha, Portugal | López-Torres & Silcox (2018) | Neustrian (PE II) | 56 Ma |
| *Arcius rougieri* | Palette, southern France | López-Torres & Silcox (2018) | Neustrian | 56 Ma |
| *Arcius lapparenti* | Meudon, Paris Basin, France | López-Torres & Silcox (2018) | Neustrian (PE II) | 56 Ma |
| *Arcius fuscus* | Fournes, southern France | López-Torres & Silcox (2018) | Neustrian | 56 Ma |
| *Acidomomys hebeticus* | SC-62, Bighorn Basin, WY | Bloch et al. (2002) | Cf-2 | 56.5 Ma |
| *Ignacius fremontensis* | Rock Bench Quarry, Bighorn Basin, WY | Secord (2008) | To-3 | 62.5 Ma |
| *Ignacius frugivorus* | Cedar Point Quarry, Bighorn Basin, WY | Secord (2008) | Ti-3 | 60 Ma |
| *Ignacius clarkforkensis* | Anthill in Rodentia zone, Bighorn Basin, WY | Secord (2008) | Cf-1 | 57 Ma |
| *Ignacius graybullianus* | WW-97S, Bighorn Basin, WY | Rose et al. (2012) | Earliest Wasatchian | 56 Ma |
| *Ignacius dawsonae* | ELS loc. 76-85, Ellesmere Island | This paper | Late Wasatchian | 52 Ma |
| *Ignacius mckennai* | ELS loc. 76-85, Ellesmere Island | This paper | Late Wasatchian | 52 Ma |

**Notes**: Geochronological estimates of FADs are based on Speijer et al. (2020). European FADs for species of *Arcius* are estimated as earliest Eocene (56 Ma), with the caveat that correlation between early Eocene sites in Portugal, southern France and the Paris Basin remains contentious (Marandat, 1997).

**References cited in S4 Table**:

Beard, K.C. & Wang, J.-W. 1995. The first Asian plesiadapoids (Mammalia: Primatomorpha). *Annals of Carnegie Museum* **64**, 1-33.

Bloch, J.I., Boyer, D.M., Gingerich, P.D. & Gunnell, G.F. 2002. New primitive paromomyid from the Clarkforkian of Wyoming and dental eruption in Plesiadapiformes. *Journal of Vertebrate Paleontology* **22**, 366-379. doi: 10.1671/0272-4634(2002)022[0366:NPPFTC]2.0.CO;2.

Clemens, W.A. & Wilson, G.P. 2009. Early Torrejonian mammalian local faunas from northeastern Montana, U.S.A. *Museum of Northern Arizona Bulletin* **65**, 111-158.

Fox, R.C. & Scott, C.S. 2011. A new, early Puercan (earliest Paleocene) species of *Purgatorius* (Plesiadapiformes, Primates) from Saskatchewan, Canada. *Journal of Paleontology* **85**, 537-548. doi: 10.1666/10-059.1.

Fox, R.C., Scott, C.S. & Rankin, B.D. 2010. *Edworthia lerbekmoi*, a new primitive paromomyid primate from the Torrejonian (early Paleocene) of Alberta, Canada. *Journal of Paleontology* **84**, 868-878. doi: 10.1666/09-072.1.

López-Torres, S. & Silcox, M.T. 2018. The European Paromomyidae (Primates, Mammalia): taxonomy, phylogeny, and biogeographic implications. *Journal of Paleontology* **92**, 920-937. doi: 10.1017/jpa.2018.10.

Marandat, B. 1997. La disparité des faunes mammaliennes du niveau MP 7 (Eocène inférieur) des domaines péri-mésogéens et nordiques. Investigation d’un provincialisme intra-européen. *Newsletters on Stratigraphy* **35**, 63-82. doi: 10.1127/nos/35/1997/63.

Rose, K.D., Chew, A.E., Dunn, R.H., Kraus, M.J., Fricke, H.C. & Zack, S.P. 2012. Earliest Eocene mammalian fauna from the Paleocene-Eocene Thermal Maximum at Sand Creek Divide, southern Bighorn Basin, Wyoming. *University of Michigan Papers on Paleontology* **36**, 1-122.

Secord, R. 2008. The Tiffanian land-mammal age (middle and late Paleocene) in the northern Bighorn Basin, Wyoming. *University of Michigan Papers on Paleontology* **35**, 1-192.

Speijer, R.P., Pälike, H., Hollis, C.J., Hooker, J.J. & Ogg, J.G. 2020. The Paleogene Period. Pp. 1087-1140 in *Geologic Time Scale 2020, Volume 2* (eds. Gradstein, F.M., Ogg, J.G., Schmitz, M.D. & Ogg, G.M.) Elsevier. doi: 10.1016/B978-0-12-824360-2.00028-0.

Szalay, F.S. 1973. New Paleocene primates and a diagnosis of the new suborder Paromomyiformes. *Folia Primatologica* **19**, 73-87. doi: 10.1159/000155534.
