# Supplementary material for: Basal Primatomorpha colonized Ellesmere Island (Arctic Canada) during the hyperthermal conditions of the early Eocene climatic optimum
Source: PLoS One. 2023 Jan 25;18(1):e0280114. doi: 10.1371/journal.pone.0280114 (PMC9876366; doi:10.1371/journal.pone.0280114)
Supplement: S4 Table — (DOCX) [file pone.0280114.s004.docx]

**S4 Table. Results of Tukey’s HSD tests for RFI and ariaDNE.**

| Pairwise comparisons | RFI | | | | ariaDNE | | | |
| --- | --- | --- | --- | --- | --- | --- | --- | --- |
|  | diff | lwr | upr | p adj | diff | lwr | upr | p adj |
| *I. dawsonae-I. clarkforkensis* | -0.111 | -0.169 | -0.053 | 0.001 | 0.009 | -0.002 | 0.021 | 0.127 |
| *I. fremontensis-I. clarkforkensis* | -0.039 | -0.106 | 0.028 | 0.403 | 0.012 | -0.001 | 0.025 | 0.083 |
| *I. frugivorus-I. clarkforkensis* | -0.046 | -0.113 | 0.020 | 0.245 | 0.011 | -0.002 | 0.024 | 0.121 |
| *I. graybullianus-I. clarkforkensis* | -0.018 | -0.100 | 0.063 | 0.983 | 0.015 | -0.001 | 0.031 | 0.068 |
| *I. mckennai-I. clarkforkensis* | -0.095 | -0.162 | -0.029 | 0.006 | 0.010 | -0.003 | 0.023 | 0.202 |
| *P. maturus-I. clarkforkensis* | -0.043 | -0.109 | 0.024 | 0.316 | 0.022 | 0.009 | 0.035 | 0.002 |
| *P. pagei-I. clarkforkensis* | -0.017 | -0.083 | 0.050 | 0.971 | 0.019 | 0.006 | 0.032 | 0.005 |
| *I. fremontensis-I. dawsonae* | 0.072 | 0.014 | 0.130 | 0.014 | 0.003 | -0.009 | 0.014 | 0.984 |
| *I. frugivorus-I. dawsonae* | 0.065 | 0.007 | 0.123 | 0.026 | 0.002 | -0.010 | 0.013 | 0.999 |
| *I. graybullianus-I. dawsonae* | 0.093 | 0.018 | 0.168 | 0.014 | 0.006 | -0.009 | 0.020 | 0.787 |
| *I. mckennai-I. dawsonae* | 0.016 | -0.042 | 0.074 | 0.950 | 0.000 | -0.011 | 0.012 | 1.000 |
| *P. maturus-I. dawsonae* | 0.069 | 0.011 | 0.126 | 0.019 | 0.012 | 0.001 | 0.024 | 0.032 |
| *P. pagei-I. dawsonae* | 0.095 | 0.037 | 0.153 | 0.002 | 0.010 | -0.002 | 0.021 | 0.107 |
| *I. frugivorus-I. fremontensis* | -0.007 | -0.074 | 0.060 | 1.000 | -0.001 | -0.014 | 0.012 | 1.000 |
| *I. graybullianus-I. fremontensis* | 0.021 | -0.061 | 0.103 | 0.967 | 0.003 | -0.013 | 0.019 | 0.990 |
| *I. mckennai-I. fremontensis* | -0.056 | -0.123 | 0.011 | 0.116 | -0.002 | -0.015 | 0.011 | 0.996 |
| *P. maturus-I. fremontensis* | -0.004 | -0.070 | 0.063 | 1.000 | 0.010 | -0.003 | 0.023 | 0.186 |
| *P. pagei-I. fremontensis* | 0.023 | -0.044 | 0.089 | 0.877 | 0.007 | -0.006 | 0.020 | 0.469 |
| *I. graybullianus-I. frugivorus* | 0.028 | -0.054 | 0.110 | 0.873 | 0.004 | -0.012 | 0.020 | 0.962 |
| *I. mckennai-I. frugivorus* | -0.049 | -0.116 | 0.018 | 0.202 | -0.001 | -0.014 | 0.012 | 1.000 |
| *P. maturus-I. frugivorus* | 0.004 | -0.063 | 0.070 | 1.000 | 0.011 | -0.002 | 0.024 | 0.128 |
| *P. pagei-I. frugivorus* | 0.030 | -0.037 | 0.097 | 0.682 | 0.008 | -0.005 | 0.021 | 0.344 |
| *I. mckennai-I. graybullianus* | -0.077 | -0.159 | 0.005 | 0.068 | -0.005 | -0.022 | 0.011 | 0.873 |
| *P. maturus-I. graybullianus* | -0.024 | -0.106 | 0.057 | 0.929 | 0.007 | -0.009 | 0.023 | 0.755 |
| *P. pagei-I. graybullianus* | 0.002 | -0.080 | 0.084 | 1.000 | 0.004 | -0.012 | 0.020 | 0.972 |
| *P. maturus-I. mckennai* | 0.053 | -0.014 | 0.119 | 0.154 | 0.012 | -0.001 | 0.025 | 0.076 |
| *P. pagei-I. mckennai* | 0.079 | 0.012 | 0.145 | 0.020 | 0.009 | -0.004 | 0.023 | 0.214 |
| *P. pagei-P. maturus* | 0.026 | -0.041 | 0.093 | 0.787 | -0.003 | -0.016 | 0.010 | 0.991 |

**Notes:** diff is the difference between group means, lwr and upr are the lower and upper end points of the interval, and p adj is the p-value adjusted for multiple comparisons.
