# Supplementary material for: Basal Primatomorpha colonized Ellesmere Island (Arctic Canada) during the hyperthermal conditions of the early Eocene climatic optimum
Source: PLoS One. 2023 Jan 25;18(1):e0280114. doi: 10.1371/journal.pone.0280114 (PMC9876366; doi:10.1371/journal.pone.0280114)
Supplement: S5 Table — (DOCX) [file pone.0280114.s005.docx]

**S5 Table. Results of the Tukey HSD tests comparing principal components by diet.**

| Pairwise Comparisons | PC1 | | | | PC2 | | | | PC3 | | | |
| --- | --- | --- | --- | --- | --- | --- | --- | --- | --- | --- | --- | --- |
|  | diff | lwr | upr | p adj | diff | lwr | upr | p adj | diff | lwr | upr | p adj |
| Arctic Fossils-Folivory | 4.334 | 3.709 | 4.959 | 0.000 | 0.250 | -0.357 | 0.857 | 0.839 | -1.125 | -1.769 | -0.482 | 0.000 |
| Mid Latitude Fossils-Folivory | 3.570 | 3.064 | 4.075 | 0.000 | 1.910 | 1.419 | 2.402 | 0.000 | 0.272 | -0.249 | 0.792 | 0.656 |
| Frugivory-Folivory | 2.056 | 1.657 | 2.456 | 0.000 | 0.873 | 0.485 | 1.261 | 0.000 | -0.088 | -0.499 | 0.324 | 0.989 |
| Hard-Object-Folivory | 3.215 | 2.842 | 3.589 | 0.000 | 0.114 | -0.249 | 0.477 | 0.943 | 0.221 | -0.164 | 0.605 | 0.557 |
| Omnivory-Folivory | 1.570 | 1.049 | 2.091 | 0.000 | 2.295 | 1.788 | 2.802 | 0.000 | -0.147 | -0.683 | 0.390 | 0.968 |
| Mid Latitude Fossils-Arctic Fossils | -0.764 | -1.441 | -0.087 | 0.017 | 1.660 | 1.002 | 2.319 | 0.000 | 1.397 | 0.700 | 2.094 | 0.000 |
| Frugivory-Arctic Fossils | -2.278 | -2.880 | -1.676 | 0.000 | 0.623 | 0.038 | 1.209 | 0.030 | 1.038 | 0.418 | 1.658 | 0.000 |
| Hard-Object-Arctic Fossils | -1.119 | -1.703 | -0.534 | 0.000 | -0.136 | -0.705 | 0.433 | 0.982 | 1.346 | 0.744 | 1.948 | 0.000 |
| Omnivory-Arctic Fossils | -2.764 | -3.453 | -2.075 | 0.000 | 2.045 | 1.376 | 2.715 | 0.000 | 0.979 | 0.269 | 1.688 | 0.002 |
| Frugivory-Mid Latitude Fossils | -1.513 | -1.991 | -1.036 | 0.000 | -1.037 | -1.501 | -0.573 | 0.000 | -0.359 | -0.851 | 0.132 | 0.284 |
| Hard-Object-Mid Latitude Fossils | -0.354 | -0.810 | 0.101 | 0.221 | -1.796 | -2.239 | -1.353 | 0.000 | -0.051 | -0.520 | 0.418 | 1.000 |
| Omnivory-Mid Latitude Fossils | -2.000 | -2.583 | -1.417 | 0.000 | 0.385 | -0.182 | 0.952 | 0.366 | -0.418 | -1.018 | 0.182 | 0.336 |
| Hard-Object-Frugivory | 1.159 | 0.825 | 1.493 | 0.000 | -0.759 | -1.084 | -0.434 | 0.000 | 0.308 | -0.035 | 0.652 | 0.105 |
| Omnivory-Frugivory | -0.486 | -0.980 | 0.007 | 0.056 | 1.422 | 0.942 | 1.902 | 0.000 | -0.059 | -0.567 | 0.449 | 0.999 |
| Omnivory-Hard-Object | -1.646 | -2.118 | -1.173 | 0.000 | 2.181 | 1.721 | 2.641 | 0.000 | -0.367 | -0.854 | 0.119 | 0.251 |

**Notes:** diff is the difference between group means, lwr and upr are the lower and upper end points of the interval, and p adj is the p-value adjusted for multiple comparisons.
