# Supplementary figures and images for: Basal Primatomorpha colonized Ellesmere Island (Arctic Canada) during the hyperthermal conditions of the early Eocene climatic optimum
Source: PLoS One. 2023 Jan 25;18(1):e0280114. doi: 10.1371/journal.pone.0280114 (PMC9876366; doi:10.1371/journal.pone.0280114)

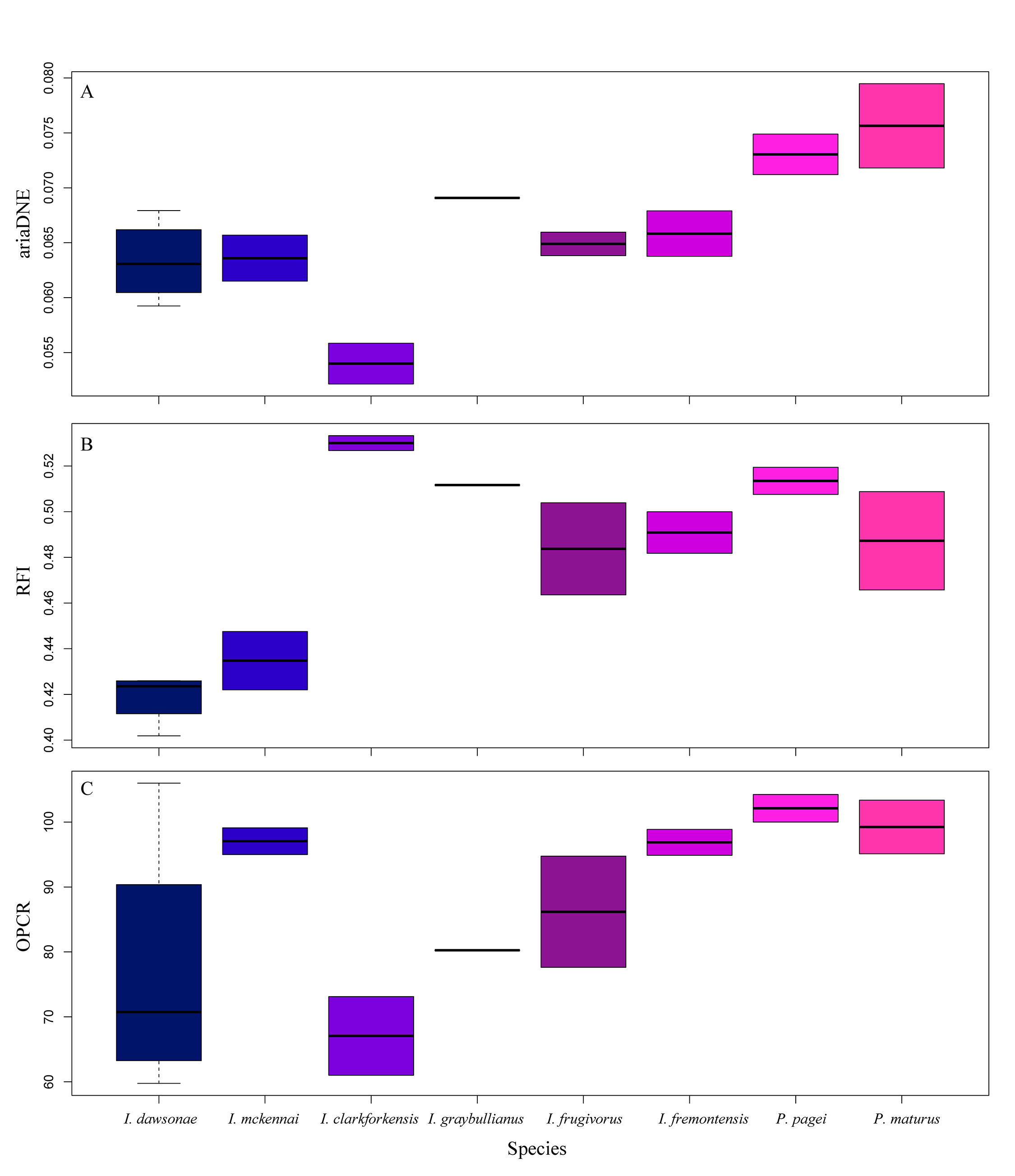

Supplement: S1 Fig — (TIF) [file pone.0280114.s011.tif]

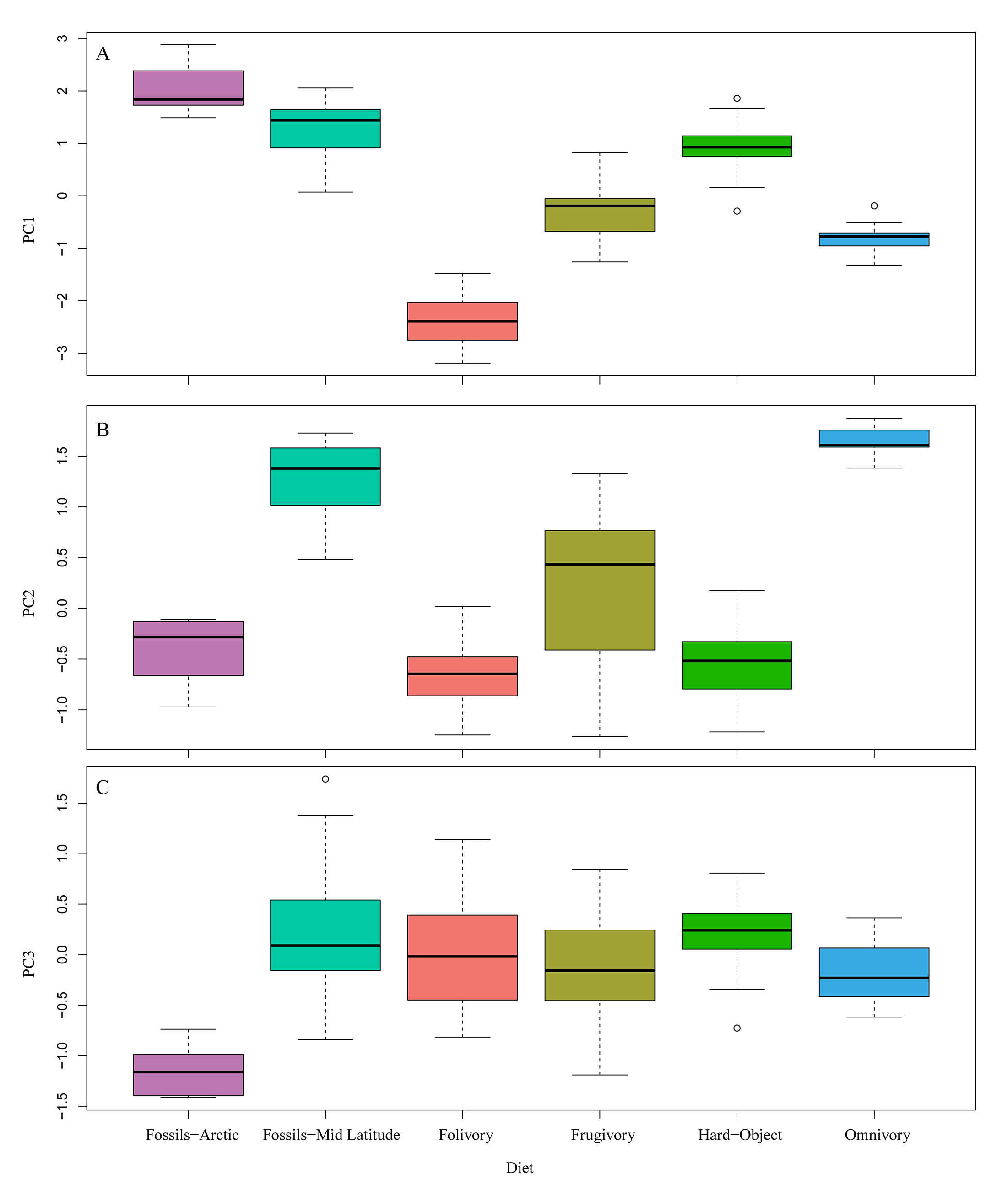

Supplement: S2 Fig — (TIF) [file pone.0280114.s012.tif]

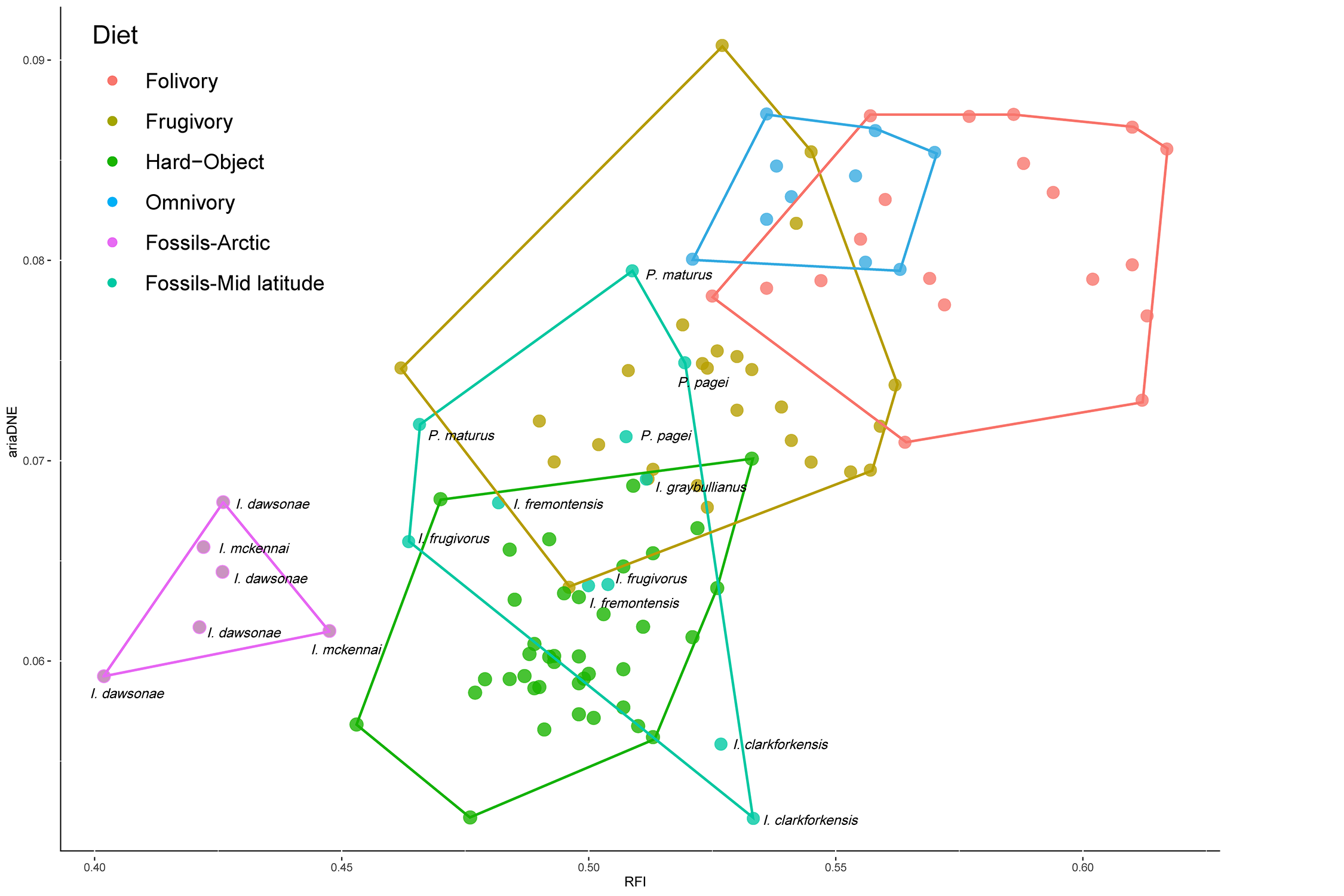

Supplement: S3 Fig — (TIF) [file pone.0280114.s013.tif]
